# Molecular Characterization of Chemosensory Protein (CSP) Genes and the Involvement of *AgifCSP5* in the Perception of Host Location in the Aphid Parasitoid *Aphidius gifuensis*

**DOI:** 10.3390/ijms25126392

**Published:** 2024-06-09

**Authors:** Jun Jiang, Jiayi Xue, Miaomiao Yu, Xin Jiang, Yumeng Cheng, Huijuan Wang, Yanxia Liu, Wei Dou, Jia Fan, Julian Chen

**Affiliations:** 1 State Key Laboratory for Biology of Plant Diseases and Insect Pests, Institute of Plant Protection, Chinese Academy of Agricultural Sciences, Beijing 100193, China; 82101209802@caas.cn (J.J.); 82101222331@caas.cn (J.X.); ymm970611@126.com (M.Y.); 18911895763@163.com (X.J.); chengymcn@foxmail.com (Y.C.); wanghuijuan0730@163.com (H.W.); 18210058781@163.com (Y.L.); 2 Key Laboratory of Entomology and Pest Control Engineering, College of Plant Protection, Southwest University, Chongqing 400716, China; douwei80@swu.edu.cn

**Keywords:** *Aphidius gifuensis*, chemosensory protein, tissue expression profile, binding affinity, molecular docking, behavioral response

## Abstract

*Aphidius gifuensis* is the dominant parasitic natural enemy of aphids. Elucidating the molecular mechanism of host recognition of *A. gifuensis* would improve its biological control effect. Chemosensory proteins (CSPs) play a crucial role in insect olfactory systems and are mainly involved in host localization. In this study, a total of nine CSPs of *A. gifuensis* with complete open reading frames were identified based on antennal transcriptome data. Phylogenetic analysis revealed that AgifCSPs were mainly clustered into three subgroups (AgifCSP1/2/7/8, AgifCSP3/9, and AgifCSP4/5/6). *AgifCSP2/5* showed high expression in the antennae of both sexes. Moreover, *AgifCSP5* was found to be specifically expressed in the antennae. In addition, fluorescent binding assays revealed that AifCSP5 had greater affinities for 7 of 32 volatile odor molecules from various sources. Molecular docking and site-directed mutagenesis results revealed that the residue at which AgifCSP5 binds to these seven plant volatiles is Tyr75. Behavior tests further confirmed that *trans*-2-nonenal, one of the seven active volatiles in the ligand binding test, significantly attracted female adults at a relatively low concentration of 10 mg/mL. In conclusion, AgifCSP5 may be involved in locating aphid-infested crops from long distances by detecting and binding *trans*-2-nonenal. These findings provide a theoretical foundation for further understanding the olfactory recognition mechanisms and indirect aphid localization behavior of *A. gifuensis* from long distances by first identifying the host plant of aphids.

## 1. Introduction

China is one of the major wheat-producing countries in the world, and Huang-Huai-Hai is the main wheat-producing area of China [1]. The wheat aphid *Sitobion miscanthi*, also widely known as *Sitobion avneae* in China, is a dominant pest of wheat that severely limits wheat production [2,3,4]. Recently, the abuse of pesticides has caused serious resistance in aphids in China, which has led to increasingly severe “3R” problems (i.e., residue, resistance, and resurgence) and environmental pollution [4,5]. Therefore, biological control as a sustainable approach for reducing the use of pesticides and even replacing them is receiving increasing attention.

It is well known that the release of *Aphidius gifuensis* has achieved satisfactory control effects against aphids such as *Myzus persicae* and *Lipaphis erysimi* in both greenhouse vegetables and open tobacco fields [6,7]. It is also the naturally predominant parasitoid of *S. miscanthi* [8] and exhibits excellent biological control efficacy, with a greater than 70% parasitism rate against wheat aphids in the Huang-Huai-Hai area of China [6,9].

Olfactory recognition is crucial to insect parasitoids such as *Aphidius* in searching for host plants, host aphids, and males for mating females [10,11,12]. Feeding by aphids induces the plant to release herbivore-induced volatiles, which can be intercepted by parasitoids while searching for aphids [10,11]. Similarly, aphids release alarm pheromones when they feel threatened, which also serves as a cue for parasitoids to target their hosts [12]. Therefore, a key scientific question worth investigating is how these two types of chemical cues attract the parasitoid *A. gifuensis*.

Odor molecules enter through tiny pores on the insect’s antennae and then pass through the hemolymph and activate odorant receptors located on the dendritic membranes of neurons [13]. Then, the ion channels formed by the receptors are opened, leading to changes in membrane potential and signal transmission to the central nervous system, ultimately triggering behavioral responses [13]. The process of insect olfactory perception involves the synergistic action of multiple olfactory proteins, such as chemosensory proteins (CSPs) [14], odorant-binding proteins (OBPs) [15], odorant receptors (ORs) [16], ionotropic receptors (IRs) [17], sensory neuron membrane proteins (SNMPs) [18], and odorant-degrading enzymes (ODEs) [13]. Different substances enter the hemolymph in different ways. For instance, carbon dioxide, acids, and amine substances can directly diffuse through the hemolymph, while most hydrophobic odor molecules, such as aromatics, heterocyclics, and alcohols, require carriers such as OBPs and CSPs to pass through the lymph to receptors [19].

CSPs are highly conserved and are found in large quantities in chemoreceptor lymph fluid [20]. As small soluble proteins with four conserved cysteines that form two disulfide bond bridges, olfactory-specific protein D (OS-D) or olfactory-specific protein D superfamily (OS-D superfamily) domains are typical sequence features of insect CSPs [14,20]. Several CSPs perform different functions in nonolfactory tissues and organs, such as in the reproductive organs of *Ophraella ommuna* and *Bemisia tabaci* [21,22], in the embryonic tissue of *Apis mellifera* [23], as carriers of pheromones in the secretory glands of *Drosophila melanogaster* [24], as carriers of carotenoids and visual pigments of *Helicoverpa armigera* [25], in insecticide resistance in *B. tabaci* [26], and in salicylic acid-mediated defense responses in *S. miscanthi* in wheat [3]. However, the olfactory function of CSPs is well known. For example, CchiCSP3 and CchiCSP5 of *Callosobruchus chinensis* are involved in the recognition of mung bean volatiles [14]. AlepCSP2 in male *Athetis lepigone* is involved in mating behavior [27]. BodoCSP1 is involved in the perception of host plant volatiles in *Bradysia odoriphaga* [28]. CSPs in some hymenoptera, such as *Polistes dominulus*, *Vespa crabro*, and *A. mellifera*, which are expressed mainly in the antennae, play roles in chemosensory perception [29]. To date, there have been few studies on the function of CSPs in parasitic wasps. EforCSP3, which is highly expressed in the female head of *Encarsia formosa*, exhibits high binding affinities to a wide range of host-related volatiles and may be involved in semiochemical reception [30]. SnocCSP4 in the male genitalia of *Sirex noctilio* involves female surface chemicals [31,32]. Elucidating the interactions between AgifCSPs and key chemical cues not only contributes to the advancement of our understanding of insect perception of hosts indirectly through the volatiles released by plants but also holds the potential to guide the design of chemical lures for aphid biocontrol and the further development of more precise control strategies. However, the functions of CSPs in *A. gifuensis* are still unclear.

*A. gifuensis* is a dominant parasitoid species in wheat fields, but the biological agents commonly lag behind in aphid outbreaks. The purpose of this study is to reveal the recognition mechanism for host plants and host aphids by *A. gifuensis* and screen some active volatile organic compounds (VOCs) to develop the attractants to improve the biological control efficacy. Therefore, the selected VOCs were mainly derived from various organic volatiles in wheat field habitats, referring to the compounds related to wheat plants and wheat aphids previously reported in published papers [11,12], including four groups of competitive ligands: (1) aphid alarm pheromone components; (2) main components of the aphid sex pheromone; (3) green leaf volatiles of wheat; and (4) aphid-induced plant volatiles. In this study, we conducted bioinformatic prediction based on both transcriptomic [11] and genomic data [33] and sequenced the full-length cDNAs of the CSPs. Next, we analyzed the expression profiles of CSP genes by quantitative real-time PCR (qRT–PCR) and targeted those genes that were highly expressed specifically in the antennae. Then, the target CSP expressed in the prokaryotic expression system was purified to screen for odor ligands with high affinity. Furthermore, molecular docking analysis and site-directed mutagenesis helped us understand how CSP binds certain ligands via certain residues. Finally, dual-choice behavioral tests were used to verify the active response of *A. gifuensis* to the odors to which AgifCSP5 could strongly bind.

## 2. Results

### 2.1. Identification and Sequence Analysis of the CSP Genes of A. gifuensis

**Figure 1:**
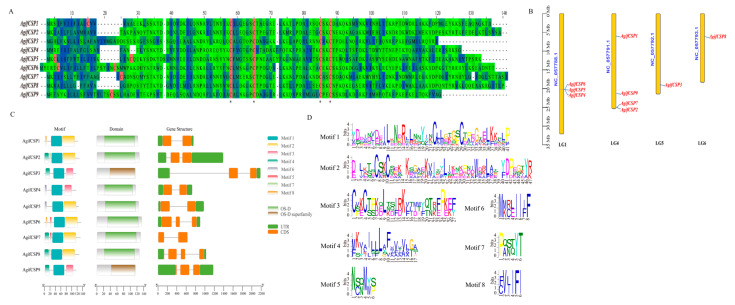
Analysis of nine AgifCSP sequences. (**A**) Sequence alignment of AgifCSPs, cysteine residues highlighted by red color and conserved cysteine residues marked with asterisks (*). (**B**) Localization of 9 *AgifCSPs* on *Aphidius gifuensis* chromosomes. (**C**) Motifs, domains and gene structure analysis of nine *AgifCSPs*. (**D**) Sequence details of eight motifs.

**Figure 2:**
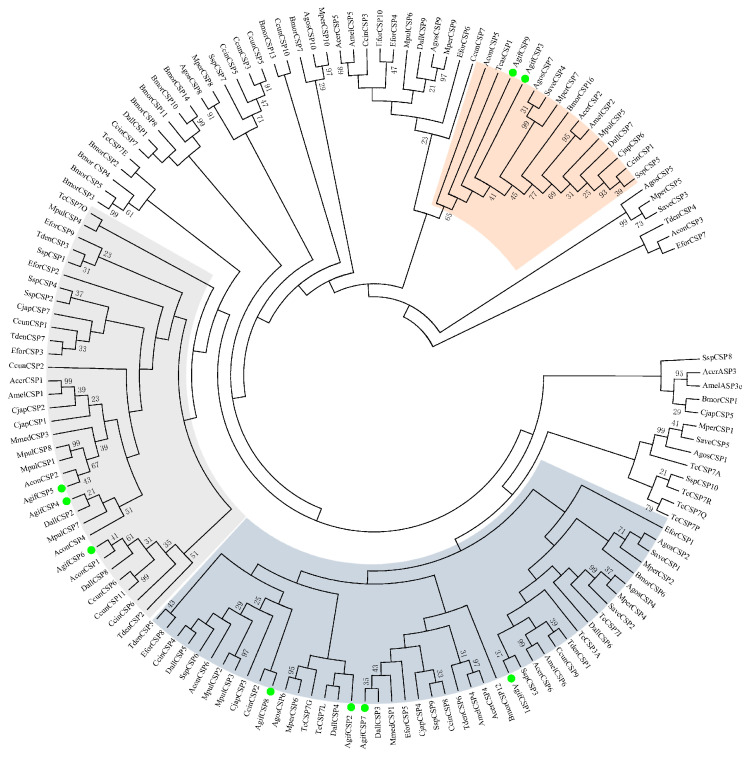
Phylogenetic tree of nine AgifCSPs of *A. gifuensis* CSPs and other insect CSPs. Seventeen species were included: *Apis mellifera*: Amel; *Apis cerana*: Acer; *Chouioia cunea*: Ccun; *Microplitis mediator*: Mmed; *Diachasma alloeum*: Dall; *Cephus cinctus*: Ccin; *Sclerodermus* sp. MQW-2015: Ssp; *Meteorus pulchricornis*: Mpul; *Aulacocentrum confusum*: Acon; *Camponotus japonicas*: Cjap; *Encarsia formosa*: Efor; *Tribolium castaneum*: Tc/Tcas; *Bombyx mori*: Bmor; *Myzus persicae*: Mper; *Aphis gossypii*: Agos; and *Sitobion avenae*: Save. The bootstrap support was calculated with 1000 rapid bootstrap replicates. Nine AgifCSPs marked with green dots, form three clusters showed by three colored shadows.

After gene prediction and cloning, we identified nine CSP genes in *A. gifuensis*. All CSP genes had complete open reading frames (ORFs) with lengths ranging from 116 to 135 amino acids. Additionally, all CSP signal peptides were 16–25 amino acids in length, and their molecular weights ranged from 10–14 kDa (Table 1). Sequence alignment revealed that all the CSPs had four conserved cysteine residues (Figure 1A, Supplementary Data S1). The results of motif prediction indicated that all the CSPs possessed motif 1, CSP3/4/9 had motif 3, and the rest had motif 2 (Figure 1C,D). All CSPs contained the typical OS-D or OS-D superfamily domains of this protein family. Gene structure analysis revealed that all CSP genes except for CSP7 had untranslated regions (UTRs) at both ends. Among them, CSP3/6/8/9 had three exons, while the others had two exons (Figure 1C). Chromosomal mapping revealed that CSP4/5/6 clustered together on the LG1 chromosome (6 genome sequences at the chromosome level named LG1 through LG6), CSP1/2/7/9 were scattered on the LG4 chromosome, and CSP3 and CSP8 were located on LG5 and LG6, respectively (Figure 1B). The phylogenetic tree was constructed by the MEGA7.0 program of all CSPs from four insect orders, including twelve Hymenoptera insects, *Apis mellifera*, *Apis cerana*, *Chouioia cunea*, *Microplitis mediator*, *Diachasma alloeum*, *Cephus cinctus*, *Sclerodermus* sp. MQW-2015, *Meteorus pulchricornis*, *Aulacocentrum confusum*, *Camponotus japonicas*, *Encarsia formosa*, and *Trichogramma dendrolimi*, a Coleopteran *Tribolium castaneum*, a Lepidoptera *Bombyx mori*, and three Hemiptera host aphids, *Myzus persicae*, *Aphis gossypii*, and *Sitobion avenae*. The amino acid sequences used to construct the phylogenetic tree are presented in Supplementary Data S2. The results showed that all AgifCSPs were mainly clustered into three homologous subgroups (Figure 2). AgifCSP1, AgifCSP2, AgifCSP7, and AgifCSP8 were grouped on one subtree; AgifCSP4, AgigCSP5, and AgifCSP6 were grouped on the second subtree; and AgifCSP3 and AgifCSP9 clustered together on the outer branches of the first two subtrees. The CSPs in aphids tended to cluster independently and did not blend with the CSPs in other orders; however, three orthologs of aphid CSPs, AgosCSP4, AgosCSP6, and AgosCSP7, in the cotton aphid *Aphis gossypii* were grouped in the subtrees adjacent to AgifCSP1, AgifCSP2, and AgifCSP3/9 (Figure 2).

#### Relative Expression of CSPs in *A. gifuensis*

**Figure 3:**
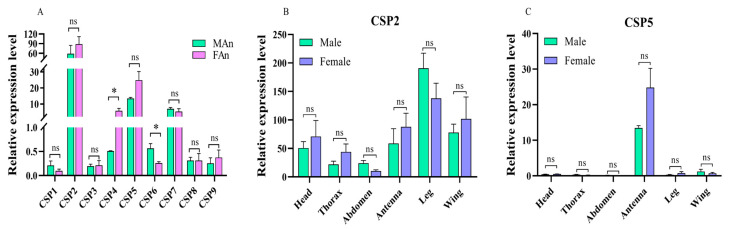
Gene expression of *AgifCSPs* in both sexes of parasitoids. (**A**), Gene expression of nine AgifCSPs in antennae; FAn and MAn are antennae of female and male adults, respectively; (**B**,**C**), Gene expression profiles of *AgifCSP2* and *AgifCSP5* in various tissues, including the head without antenna, thorax, abdomen, antenna, leg, and wing of *A. gifuensis*; ‘*’ indicates that gene expression was significantly different between males and females at *p* < 0.05 according to a *t* test, and ‘ns’ indicates no difference between the sexes.

The expression profiles of nine CSP genes in the antennae of *A. gifuensis* were analyzed (Figure 3A). *AgifCSP2* and *AgifCSP5* were the two CSPs with the highest expression levels. The expression levels were not significantly different when stratified by sex (*AgifCSP2*, *F* = 0.133, *t* = −0.839, *p* = 0.449; *AgifCSP5*, *F* = 4.789, *t* = −2.093, *p* = 0.104). AgifCSP7 was also expressed at comparatively high levels in both males and females. There were significantly more AgifCSP4-positive females than males (*F* = 5.683, *t* = −3.276, *p* = 0.031). Conversely, females expressed significantly lower levels of AgifCSP6 than males (*F* = 1.593, *t* = 2.971, *p* = 0.041). Other CSPs were expressed at generally low levels and showed no sex specificity in the antennae. Furthermore, the expression patterns of *AgifCSP2* and *AgifCSP5* in tissues and organs of both sexes were analyzed (Figure 3B,C). The results showed that *AgifCSP2* was widely expressed throughout the body, with the highest expression in the legs. *AgifCSP5*, however, was specifically expressed in the antennae of both sexes.

### 2.2. Purification and Expression of AgifCSP5

**Figure 4:**
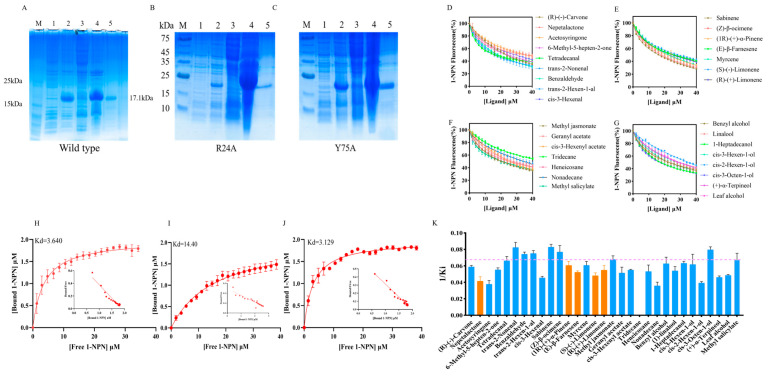
Analysis of AgifCSP5 protein expression, purification, and binding profiles. (**A**–**C**) Wild-type, R24A, Y75A protein purification, M: 180 kDa protein molecular marker; Lane 1: Noninduced AgifCSP5-pET30a; Lane 2: induced AgifCSP5-pET30a; Lane 3: induced AgifCSP5 supernatant lysate; Lane 4: inclusion body; Lane 5: purified protein. (**D**–**G**) Competitive binding curves of AgifCSP5 to compounds. (**D**) Ketones and aldehydes. (**E**) Alkenes. (**F**) Esters and alkanes. (**G**), Alcohols. (**H**–**J**) Binding curves and scatter plots (insert) of the wild type, R24A, and Y75 of AgifCSP5 to 1-NPN. (**K**) 1/Ki of all ligands to AgifCSP5; the blue bars indicate plant volatiles, the orange bars indicate aphid pheromones, and the red dashed lines indicate strong binding affinity.

*AgifCSP5*, a CSP highly expressed specifically in the antennae, was chosen for further investigation of its ligand binding spectrum. We first fused *AgifCSP5* into the expression vector PET30a and expressed it for purification. The purified protein was obtained at a concentration of 0.55 mg/mL, with a molecular weight of approximately 17.1 kDa (Figure 4A), consistent with the predicted results.

### 2.3. Fluorescent Competitive Binding Assays of AgifCSP5 with Ligands

After obtaining the purified protein, fluorescence competitive ligand binding tests were conducted to measure the binding affinity of the protein for ligands. The binding constant of AgifCSP5 with 1-NPN was determined to be 3.640, and the Scatchard plots are shown in Figure 4H. AgifCSP5 exhibited strong affinity (Ki < 15 μM) for volatile aldehydes, including *trans*-2-nonenal (Ki = 12.27 μM), benzaldehyde (Ki = 13.49 μM), and *trans*-2-hexen-1-al (Ki = 13.38 μM); alkenes, including sabinene (Ki = 12.12) and (*Z*)-β-ocimene (Ki = 13.31); esters, including methyl jasmonate (Ki = 14.86 μM); and alcohols, including *cis*-3-octen-1-ol (Ki = 12.61 μM) (Figure 4D–G and Table 2). AgifCSP5 exhibited moderate or weak affinity for tetradecanal (Ki = 15.26 μM), 1-heptadecanol (Ki = 15.85 μM), the aphid alarm pheromone (*E*)-β-farnesene (Ki = 19.15 μM), and the aphid sex pheromone nepetalactone (Ki = 25.00 μM) (Figure 4D,E and Appendix B). The reciprocals of the binding constants for all the compounds are presented in Figure 4K.

### 2.4. Molecular Docking of AgifCSP5 with Ligands

**Figure 5:**
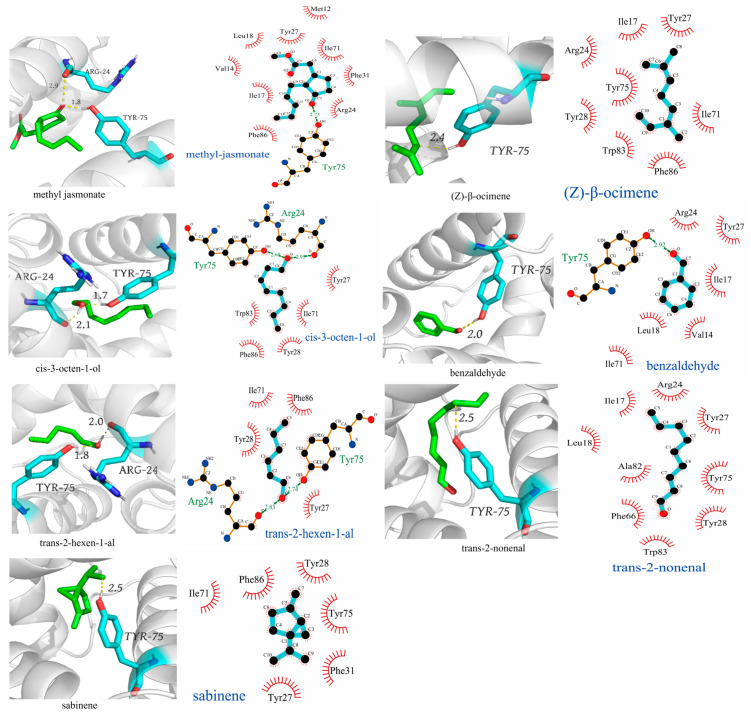
3D and 2D structures of AgifCSP5 interacting with ligands.

The molecular docking of nine compounds that had strong binding affinities (Ki < 16 μM) with AgifCSP5 was analyzed using AutoDock 4.2.6 software, and the results were visualized using LigPlus and PyMOL. The amino acid residues Val4, Met12, Ile17, Ile18, Arg24, Tyr27, Tyr28, Phe31, Phe45, Ile71, Tyr75, and Phe86 collectively formed a hydrophobic amino acid binding pocket (Figure 5). All the compounds were docked in this pocket. The results showed that *cis*-3-octen-1-ol and *trans*-2-hexen-1-al formed two hydrogen bonds (H bonds) with Arg24 and Tyr75. Methyl jasmonate and benzaldehyde interacted with one key residue of Tyr75 via H-bonding. Although (*Z*)-β-ocimene, *trans*-2-nonenal, and sabinene did not form H bonds with AgifCSP5 according to the 2D diagram, the 3D diagram showed that the distance between the three chemicals and the Tyr75 residue was no more than 3 Å (Figure 5). The 3D diagram showed that the distance between (*Z*)-β-ocimene, *trans*-2-nonenal, and sabinene 3 chemicals and the Tyr75 residue were 2.4 Å, 2.5 Å, and 2.5 Å, with no more than 3 Å (Figure 5), were all longer than Methyl jasmonate and benzaldehyde (1.8 Å and 2.0 Å in the 3D diagram), that both of them could form an H bond with Tyr75.

### 2.5. Site-Directed Mutagenesis and Binding Characteristics

**Figure 6:**
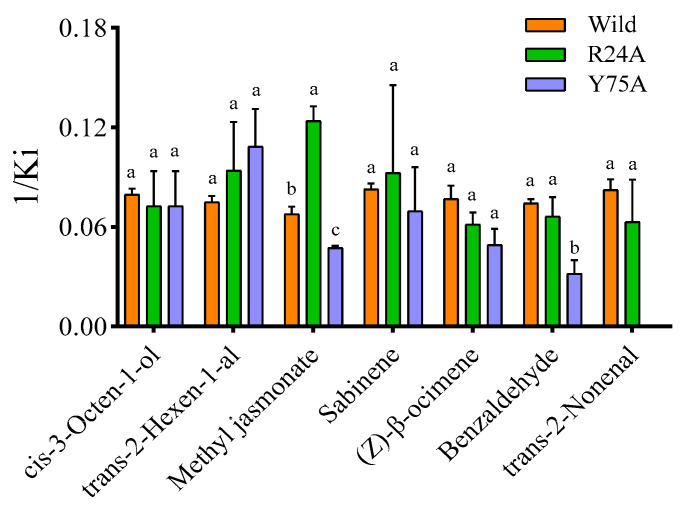
1/Ki of ligands to mutants (R24A and Y75A) of AgifCSP5. The error bars represent the standard error of the mean (mean ± SE), and values followed by different letters are significantly different at *p* < 0.05 according to the LSD test.

Two predicted key residues of AgifCSP5, namely, Arg24 and Tyr75, were site-directed mutated to alanine to simplify the protein structure, and the mutants were named R24A and Y75A, respectively. The purity and quality of the purified proteins determined by SDS-PAGE are shown in Figure 4B,C. The binding constants of the R24A and Y75A proteins with 1-NPN were determined to be 14.400 μM and 3.129 μM, respectively, and the Scatchard plots are shown in Figure 4I,J. The results indicated that Y75A lost its ability to bind to *trans*-2-nonenal, benzaldehyde, tetradecanal, and methyl jasmonate (Table 2 and Figure 6). However, R24A increased the binding affinity to methyl jasmonate and tetradecanal and had no difference from the wild type for the other seven compounds.

### 2.6. Behavior Response of A. gifuensis to Volatiles

**Figure 7:**
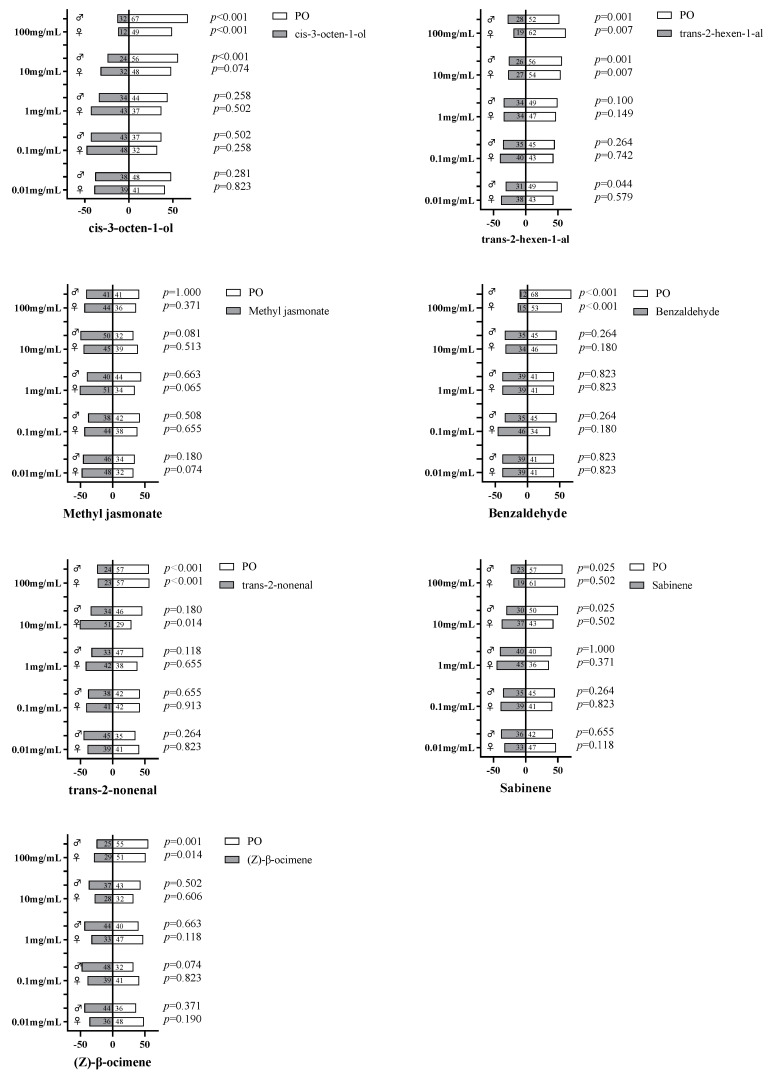
Behavioral responses of *A. gifuensis* to various volatiles. The chi-square test (χ2) was performed to detect significant differences in behavioral responses between compounds and paraffin oil (PO).

To further investigate the volatile behavioral activity of AgifCSP5 against *A. gifuensis*, Y-tube olfactometry assays were conducted (Figure 7). The results indicated that most volatiles, such as *cis*-3-octen-1-ol, *trans*-2-hexen-1-al, benzaldehyde, *trans*-2-nonenal, sabinene, and (*Z*)-β-ocimene at 100 mg/mL, significantly repelled *A. gifuensis* adults at high concentrations (>10 mg/mL). At 10 mg/mL, *trans*-2-hexen-1-al also obviously repelled *A. gifuensis*. The two compounds, *cis*-3-octen-1-ol and sabinene, significantly repelled only males at 10 mg/mL but repelled both sexes at 100 mg/mL. Surprisingly, we found that *trans*-2-nonenal significantly attracted only female adults at 10 mg/mL but repelled both sexes at 100 mg/mL.

## 3. Discussion

CSPs play a crucial role in chemoreception by recognizing, binding, and transporting hydrophobic odor molecules in insects [20]. In recent years, binding affinity assays have been successfully used to investigate the affinity of CSPs for odor ligands, and further combination with behavioral experiments has been helpful for screening behaviorally active compounds [27,30]. However, related research is more challenging in the context of tiny hymenopteran insects such as *A. gifuensis*. In this study, we identified and characterized the molecular features and expression profiles of nine CSPs from 12 previously reported CSPs that were preliminary predicted by transcriptomic annotation [34]. Three of them were ultimately excluded due to the lack of four conserved cysteine residues and the OS-D domain. Previous studies have reported that there are 8 CSPs in *M. pulchricornis* [35], 11 in *Cotesia vestalis* [36], 10 in *E. formosa* [37], and 11 in *C. cunea* [38]. These findings suggested that hymenopteran insects possess similar numbers of CSPs. All AgifCSPs were mainly clustered into three homologous subgroups. The amino acid sequences of chemosensory proteins (CSPs) of other hymenoptera parasitoids than *A. gifuensis* were downloaded from the uniProt database. Moreover, CSPs from *A. mellifera*, *A. cerana,* and *C. japonicas* which are model species, were also included. In addition, CSPs from *C. cinctus*, a phytophagous hymenopteran on wheat, were selected because they share partially overlapping habitats when *A. gifuensis* colonizes in the wheat fields.

Antennae are crucial organs by which insects perceive external information; thus, highly expressed olfactory genes are often considered key target genes for further functional analysis. In this study, we selected *AgifCSP5*, which is highly expressed in the antennae of both male and female parasitoids. Further analysis showed that although the expression level of *AgifCSP5* in female antennae was higher than that in male antennae, there was no statistically significant difference, which may be due to the large variation in individual olfactory protein expression level in females at high abundance. Moreover, we also noted that on the phylogenetic tree, EforCSP3 was grouped on the same subtree as AgifCSP5, which has been previously reported to be involved in the recognition of host plant volatiles in another parasitoid, *E. formosa* [30]. Therefore, we hypothesized that AgifCSP5 may be involved in host or mate location behaviors in *A. gifuensis*. Fluorescence competitive binding assays revealed the affinities of AgifCSP5 for various plant volatiles, such as *trans*-2-hexen-1-al, *trans*-2-nonenal, benzaldehyde, sabinene, (*Z*)-β-ocimene, methyl jasmonate, and 1-heptadecanol. Furthermore, site-directed mutagenesis revealed that Tyr75 was involved in the interaction of AgifCSP5 with plant compounds. Notably, among the nine active molecules screened from the ligand binding test, *trans*-2-nonenal attracted *A. gifuensis* and elicited a strong positive behavioral response.

Chromosomal mapping revealed that CSP4, CSP5, and CSP6 were next to each other on the same chromosome, indicating typical gene duplication from a common ancestral gene. Not surprisingly, phylogenetic analysis revealed that the three genes clustered in the same subtree: AgifCSP4 and AgifCSP5 were closer, while AgifCSP6 was located on an outer branch. The results of chromosome localization also showed that AgifCSP3 and its paralog, AgifCSP9, were separately located on different chromosomes. In addition, CSP8 was separated from its paralogs, CSP1/2/7/9, and located independently on another chromosome. See Supplementary Data S1 for sequencing details. These results suggest that the expansion of CSP family members from a few ancient CSPs may have been completed even before chromosome division and then gradually differentiated into different functions through the ages of evolution. Various expression patterns could be discovered in the paralogs; for example, in contrast to *AgifCSP5*, *AgifCSP4* is highly expressed in only female antennae, whereas the expression of AgifCSP6 is significantly lower in the antennae of both sexes. Such a wide difference in expression patterns suggests that CSP4, CSP5, and CSP6 may have undergone specific functional differentiation to adapt to the acquisition of certain environmental information by olfaction. More studies will be needed to confirm this possibility.

Reverse chemical ecology studies involving computer-aided virtual screening and the heterologous expression of candidate proteins in prokaryotic systems have been conducted to analyze these key target genes [14,39]. To investigate the function of AgifCSP5, a total of 30 compounds, including plant volatiles and aphid pheromones, were subjected to fluorescence competitive binding assays at pH 7.4. AgifCSP5 exhibited strong binding affinity (Ki < 15 μM) to plant volatiles such as benzaldehyde, *trans*-2-nonenal, *trans*-2-hexen-1-al, sabinene, (*Z*)-β-ocimene, methyl jasmonate, *cis*-3-octen-1-ol, tetradecanal, and 1-heptadecanol, of which *trans*-2-nonenal was further proven to be attractive to *A. gifuensis* at a concentration of 10 mg/mL and repelled at a higher concentration of 100 mg/mL. Previous studies have shown that McinOBP1 in *Macrocentrus cingulum* could bind to *trans*-2-nonenal [40]. Moreover, benzaldehyde, *trans*-2-hexen-1-al, sabinene, (*Z*)-β-ocimene, and *cis*-3-octen-1-ol all showed repellent effects at a high concentration of 100 mg/mL but did not show any repelling or attracting activity at low concentrations. Moreover, AgifCSP5 only weakly binds to the aphid sex pheromone nepetalactone and the alarm pheromone (*E*)-β-farnesene. These results indicate that AgifCSP5 is a CSP with an affinity for plant volatiles.

As important chemical cues for third-trophic-level predators, host-released pheromones play a crucial role in the host localization of natural enemies. However, in this study, AgifCSP5 showed only weak binding affinities to host aphid pheromones (EBF and nephelactone), suggesting the involvement of other proteins in the recognition of host pheromones. For example, we previously reported that AgifOBP6, which is highly expressed in the antennae, may play a role in the recognition of the aphid alarm pheromone EBF in *A. gifuensis* [11]. In addition, for *M. mediator*, MmedCSP3 exhibited high binding affinities (Ki = 17.24–18.77 μM) to host pheromones such as *cis*-11-hexadecenyl aldehyde (Z11-16: Ald), *cis*-11-hexadecanol (Z11-16: OH), and *trans*-11-tetradecenyl acetate (E11-14: Ac), facilitating host localization [41]. These findings indicated that other CSPs could also be involved in the recognition of host pheromones.

Molecular docking results showed that AgifCSP5 formed a hydrophobic binding pocket, and several key amino acid residues in the pocket were predicted to participate in ligand binding. Most AgifCSP5-affinity odor ligands could form strong hydrogen bonds with Arg24 and Tyr75, which are located in the second and fourth α-helices, respectively, with all hydrogen bond (H bond) distances less than 3 Å. A 3D diagram was constructed to illustrate the interactions of *trans*-2-nonenal, sabinene, and (*Z*)-β-ocimene with Tyr75 with distances less than 3 Å, although H-bonds were not visualized in the 2D structure. Furthermore, site-directed mutagenesis demonstrated that Tyr75 is a vital amino acid residue of AgifCSP5 involved in binding to plant volatiles. Similar studies have been widely used to reveal the binding mechanisms of olfactory proteins to plant volatiles. For example, Glu130, a key residue of McinOBP1 in *M. cingulum*, binds to the same volatile *trans*-2-nonenal [40]. The T9A mutation in the rGmolOBP2 protein of *Grapholita molesta* reduces its binding affinity to the pheromone dodecanol [42]. In *B. odoriphaga*, the V48A and T68A mutations in BodoCSP1 significantly reduce the binding affinity to plant volatiles compared to that of wild-type BodoCSP1 [28]. However, we found increased affinities of R24A for methyl jasmonate and no difference in the affinities of R24A for the other compounds compared with those of the wild-type AgifCSP5. One possible reason is that local structural changes could occur when the Arg residue at position 24 of AgifCSP5 is replaced by Ala. Finally, Tyr75 rather than Arg24 is an important residue for the binding of AgifCSP5 to these plant volatiles.

In the present study, benzaldehyde and *trans*-2-hexenal strongly repelled adults at a high concentration of 100 mg/mL. Similar effects of 10^−2^ (*v*/*v*) benzaldehyde and 10^−3^ (*v*/*v*) *trans*-2-hexenal were also reported recently [43]. Interestingly, the lower concentration of *trans*-2-nonenal (10 mg/mL) significantly attracted *A. gifuensis* females. This effect was also observed at a low concentration of 1 µg/10 µL in the herbivore beetle *Aulacophora foveicollis* [44]. If we could define the response of parasitoids to high concentrations of plant volatiles as indicative of when the parasitoids are closer to the crop, then the response to low concentrations can be understood as indicative of the response when the parasitoids are searching for crops from a distance. The hunting processes of natural enemies may be divided into long- and short-distance hunting. Among them, host plant volatiles mainly help in locating herbivore-infested plants from long distances [45]. Herbivore pheromones, or body surface info-chemicals, are key chemical clues for natural enemies to target their prey at close ranges [46]. At first, parasitoids are attracted by relatively low concentrations of pest-induced crop volatiles at a distance, and then they arrive at aphid-infested crops. Even though they are immersed in relatively high concentrations of pest-induced crop volatiles, they are no longer attracted to them and begin to target their host aphids by detecting the info-chemicals released from the aphids. These five behaviorally active compounds, whether repellent or attractive, may act together in this shift of concern in parasitoids from aphid-infested plants to the aphids themselves.

In summary, *AgifCSP5*, a CSP specifically expressed in antennae, showed binding affinities for widely herbivore-induced plant volatiles and opposite effects of its mutagenesis. Furthermore, volatiles with high affinity for AgifCSP5 exhibit complex olfactory behavioral activities. AgifCSP5 may play a role in the long-distance location of aphid-infested plants in *A. gifuensis*. The release of screened volatiles in wheat fields will reduce the lag behind the effect of the artificial release of *A. gifuensis* in controlling the aphid outbreak. However, the electrophysiology response of *A. gifuensis* to active plant volatiles needs to be further elucidated by EAG, and the recognition mechanism of *A. gifuensis* needs to be further validated by RNAi. In addition, whether there is a relatively low expression of other AgifCSPs in conjunction with AgifCSP5 to bind these screened active volatiles and whether these volatiles have good attraction to *A. gifuensis* in wheat fields still needs further study in the future.

## 4. Materials and Methods

### 4.1. Insects and Tissue Collection

*A. gifuensis* was originally collected from mummies of *M. persicae* on tobacco plants in Kunming, Yunnan Province, China (E 102°46′16″, N 25°7′42″). A colony was established on *S. miscanthi* with nylon mesh (40 cm × 40 cm × 40 cm) on wheat (*T. aestivum*) ‘AK58’ in the laboratory of the Institute of Plant Protection of the Chinese Academy of Agricultural Sciences (Beijing, China) (E 116°17′30″, N 40°1′44″) under the following conditions: 25 ± 1 °C, 50 ± 5% relative humidity, and a photoperiod of 16L:8D. Single mummies were placed in *petri* dishes containing moistened filter paper, and adults were fed a 15% glucose solution. Tissues or organs (antennae, heads without antennae, thoraxes, abdomens, legs, and wings) of both three-day-old male and female adults were separately collected for total RNA extraction. The tissue or organs for each sample were dissected from 50 adults, collected from another two replicates, and stored at −80 °C.

### 4.2. Total RNA Extraction and cDNA Preparation

Total RNA was extracted using a micro total RNA extraction kit (Genstone Biotech, Beijing, China) following the manufacturer’s instructions. RNA purity and concentration were checked using a Nanodrop ND-1000 spectrophotometer (NanoDrop Products, Wilmington, DE, USA). RNA degradation and contamination were monitored on 2% agarose gels. cDNAs were synthesized using HiScript^®^III All-in-one RT SuperMix (Vazyme, Nanjing, China) following the manufacturer’s protocol.

### 4.3. Sequence Verification and Analysis of AgifCSPs

The sequences of *AgifCSPs*, identified from the transcriptome of *A. gifuensis* antennae, were previously published by our team [34]. The genome was downloaded from GenBank (accession number GCA_014905175.1), and the complete sequence of AgifCSP5 was obtained (accession number XP_044006396). The ORFfinder tool of NCBI (https://www.ncbi.nlm.nih.gov/orffinder/ (accessed on 3 January 2022)) was used to query the ORF lengths of the sequences with the default parameter. Polymerase chain reaction (PCR) was conducted with an Eppendorf Mastercycler^®^ gradient PCR machine using 2×Taq MasterMix (Novoprotein, Suzhou, China) to amplify all the CSP genes, with the resulting antennal cDNA serving as a template. The initial denaturation step was 95 °C for 2 min, followed by 36 cycles of 95 °C for 1 min, 60 °C as the annealing temperature for 45 s, and 72 °C for 1 min, and a final extension at 72 °C for 10 min. The PCR products were electrophoresed on 1.5% agarose gels and stained with 4SGelred (Sangon Biotech, Shanghai, China) to ensure that the amplified products were correct. All the sequences were verified by sequencing. Because all CSPs typically have an N-terminal signal peptide that aids in localization, in the present study, the signal peptides were then predicted using the SignalP-6.0 online tool (https://services.healthtech.dtu.dk/services/SignalP-6.0 (accessed on 22 March 2024)). The isoelectric point and molecular weight of AgifCSP5 were determined using the SWISS-PROT program (ExPASy server: https://web.expasy.org/protparam/ (accessed on 10 March 2022)), and the relevant results are shown in Table 1. The domains of AgifCSPs were searched on the NCBI-CDD server (https://www.ncbi.nlm.nih.gov/Structure/bwrpsb/bwrpsb.cgi (accessed on 15 April 2022)) with an E-value < 0.01; motifs were predicted on MEME (https://meme-suite.org/meme/tools/meme (accessed on 16 April 2022)) with motif number set to 8, and both of sets results were visualized by TBtools [47]. The chromosomal mapping analysis was also conducted using TBtools.

The sequence similarity of AgifCSP5 to other insect species was determined via UniProt (https://www.uniprot.org/ (accessed on 6 August 2022)), and amino acid sequences were aligned through Clustal Omega (https://www.ebi.ac.uk/Tools/msa/clustalo/ (accessed on 7 August 2022)) and visualized by Jalview [48] (Supplementary Data S2). A phylogenetic tree based on the maximum likelihood method was constructed using the MEGA7.0 program [49] with the LG+ model, and node support was assessed using a bootstrap procedure with 1000 replicates.

### 4.4. Spatial Expression Pattern of AgifCSPs

The expression profiles of AgifCSPs in the antennae of both male and female adults were analyzed by RT–qPCR using an ABI 7500 real-time PCR system (Applied Biosystems, Fosters City, CA, USA). The two CSPs with the highest expression in the antennae were chosen for analysis of their expression profiles in tissues or organs of both sexes. The primers used were designed (Appendix A) with the online tool Primer3Plus (https://www.bioinformatics.nl/cgi-bin/primer3plus/primer3plus.cgi (accessed on 22 October 2022)). We used the β-actin and NADH genes [11] as two internal controls for the normalization of AgifCSP5 expression. Each reaction contained 10 μL of 2×Taq Pro Universal SYBR qPCR Master Mix (Vazyme, China), 1 μL of cDNA, 0.5 μL of gene-specific primers, and 8 μL of sterilized ultrapure water. PCR was performed at 95 °C for 30 s, followed by 40 cycles at 95 °C for 10 s and at 60 °C for 30 s. The amplification efficiency was calculated by constructing a standard curve from five 10-fold serial dilutions of the template concentration. The resulting amplification efficiencies of 95–105% were used for subsequent data analysis. To determine the mRNA expression levels in *A. gifuensis*, the comparative 2^−ΔΔCT^ method [50] was performed as described by Livak and Schmittgen (2001).

### 4.5. Construction of Recombinant Plasmid

*AgifCSP5* was chosen for further characterization because its high expression in the antennae implies that it is involved in olfactory detection, in contrast to the more broadly expressed AgifCSP2, which may be involved in other chemosensory or nonchemosensory functions. *AgifCSP5* was amplified by PCR with a forward primer (5′-CGGGATCCCAGGAAAAATATTCAGATAAATATGATAG-3′) containing a BamH I restriction site and a reverse primer (5′-CCAAGCTTTTATTTTCTTGATGGAGTTACAATATT-3′) containing a Hind III restriction site. The PCR product was ligated into a pTOPO-TA/Blunt vector (Aidlab, Beijing, China), and the resulting product was subsequently transformed into *Escherichia coli* (DH5α). PCR-confirmed positive clones were subsequently grown in Luria-Bertani (LB) medium supplemented with kanamycin (50 μg mL^−1^) and subsequently sequenced. The pTOPO-TA/Blunt plasmid containing the target sequence was then excised, subcloned, and inserted into the bacterial expression vector pET30a with the Nde I and EcoR I restriction sites, and the recombinant plasmids were transformed into the *E. coli* BL21(DE3) strain. A sequence-verified plasmid was used to obtain mature proteins.

### 4.6. Expression and Purification of Recombinant Protein

The methods of expression and purification of recombinant proteins were referred in the previous article published by Jiang et al. in 2023, with a minor modification [11]. The positive clone carrying the sequence-verified plasmid was cultured in 5 mL of LB medium supplemented with 50 μg mL^−1^ kanamycin sulfate, shaken (220 rpm) for approximately 3.5 h at 37 °C, diluted in 2000 mL of LB medium, and cultured until an OD_600_ of 0.6 was reached. The culture was further incubated with isopropyl-1-thio-beta D-thiogalactopyranoside (IPTG: 1 mM) at 28 °C for 12 h. The expressed protein was obtained from the inclusion body after ultrasonication and centrifugation. The inclusion body protein was dissolved in 50 mM Tris-HCl buffer (pH = 7.4) with 5 mL of 6 M guanidine hydrochloride (GuHCl) and subsequently incubated for one hour in 10 mM DDT. Four milliliters of a mixture of 100 mM cystine, 500 mM NaOH, and 5 mM cysteine were added to renature the protein. The protein solution was dialyzed in 50 mM Tris-HCl buffer at pH 7.4 three times for one hour each, followed by dialysis overnight before purification. Anion-exchange chromatography with a RESOURCE Q15 HP column (GE HEALTH CARE, Chicago, IL, USA) and gel filtration [Superdex 75 10/300GL column (GE HEALTH CARE, USA)] were used to purify the proteins. The column was preequilibrated with buffer A (20 mM Tris-HCl, pH 8.5), and the protein solution was allowed to pass through the column and then washed and eluted with buffer B (20 mM Tris-HCl, 1 M NaCl, pH 8.5). The collected protein was analyzed by 15% SDS-PAGE, subsequently dialyzed as described above, and ultracentrifuged for 45 min using a 3-kDa Millipore column at 4 °C and 5000 rpm. The concentration of purified AgifCSP5 was determined by a BCA Protein Assay Kit (Cat# PC0020, Solarbio, Beijing, China), and the purified protein solution was stored in Tris-HCl (50 mM, pH 7.4) buffer at −80 °C until use.

### 4.7. Fluorescence Competitive Binding Assay

The candidate VOCs were mainly derived from various organic volatiles in wheat field habitats, referring to the compounds related to wheat plants and wheat aphids previously reported in a published paper by Jiang et al. in 2022 and 2023 [11,12], including four groups of ligands: (1) aphid alarm pheromone components; (2) aphid sex pheromone; (3) green leaf volatiles of wheat; and (4) aphid-induced plant volatiles. A total of 32 ligands were tested with fluorescence competitive binding assays to assess the binding affinity of AgifCSP5 using N-phenyl-1-naphthylamine (1-NPN) as a fluorescent probe. The classes, CAS numbers, and purities of the chemicals used in these assays are listed in Appendix B. A Lengguang 970CRT spectrofluorimeter (Shanghai Jingmi, Shanghai, China) equipped with a 1 cm light path fluorimeter quartz cuvette was used to record the fluorescence intensity at room temperature with the following parameters: the excitation wavelength was set at 337 nm, the emission light wavelength was recorded at 350–500 nm, the high-speed scan mode was used, and the slit width was 10 nm. To determine the binding affinity of 1-NPN for AgifCSP5 proteins, a 2 µM protein solution diluted with 50 mM Tris-HCl at pH 7.4 was titrated with aliquots of the 1 mM 1-NPN stock solution to final concentrations ranging from 2–40 μM. Ligands bound to AgifCSP5 have a protein:ligand ratio of 1:1. Three independent repeats were carried out for all measurements. The binding affinity of AgifCSP5 for all ligands was calculated by the formula Ki = [IC50]/(1 + [1 − NPN]/K_1-NPN_), where the IC50 is defined as the concentration of a competitor that caused a 50% reduction in fluorescence intensity, K_1-NPN_ is the dissociation constant (Kd) of the AgifCSP5/1-NPN complex, and [1-NPN] is the free concentration of 1-NPN. The volatiles that exhibited strong affinity (Ki < 15 μM) with AgifCSP5 were chosen for further analysis.

### 4.8. 3D Structural Modeling and Molecular Docking

The *AgifCSP5* sequence was queried against a protein database (https://www.rcsb.org (accessed on 15 November 2023)) and uploaded to the SWISS-MODEL homology modeling online server (http://swissmodel.ExPASy.org/ (accessed on 15 November 2023)) for the construction of 3D structures. The three-dimensional model of AgifCSP5, which was predicted by AlphaFold2 with a GMQE value of 0.93 and an identity of 100%, was downloaded from the AlphaFold Protein Structure Database (https://AlphaFold.ebi.ac.uk (accessed on 16 November 2023 & 18 November 2023)) with accession no. AF-A0A3Q9ELG9-F1. Ligands for docking studies were selected based on the florescence competitive binding assay and downloaded from PubChem (https://pubchem.ncbi.nlm.nih.gov/ (accessed on 20 November 2023)). Specifically, ligands with a Ki value < 15 μM (Appendix B) were chosen. The docking input files were generated by Autodock MGLTools (version 1.5.6) software, and the docking process was performed by Autodock4.2.6 [51] with the Lamarckian Genetic Algorithm and default parameters for the other steps. The top 10 docking poses ranked by binding energy were analyzed with Open-Source PyMOL^TM^ (Schrödinger, LLC, New York, NY, USA) and LigPlot^+^ v2.2 [52].

### 4.9. Site-Directed Mutagenesis

The mutagenesis primers for the targeted key amino acid residues were designed online (https://crm.vazyme.com/cetool/singlepoint.html (accessed on 24 December 2023)) according to the protocols of the Mut Express II Fast Mutagenesis Kit V2 (Vazyme, China) (Appendix B). Mutations were obtained by using Phanta Max Superfidelity DNA Polymerase with recombinant Plasmid pET-30a/AgifCSP5 plasmid DNA as a template. The plasmids of the mutants were verified by DNA sequencing. The mutated proteins were then expressed and purified, as described above. After protein concentration determination, the binding affinities of the mutants with ligands that previously bound well to the AgifCSP5 protein were verified using fluorescence competitive binding assays.

### 4.10. Y-Tube Olfactometer Assay

The behavioral responses of *A. gifuensis* to volatiles were tested using a Y-tube olfactometer (3.5 cm in diameter, arms 20 cm in length, and a stem 20 cm in length). The assay method was described by Guo et al. [16], and minor improvements were made. The incoming air at a constant flow (300 mL/min) was first filtered using allochroic silica gel and activated carbon and then humidified with ultrapure water. The test compounds were serially diluted with paraffin oil to concentrations of 0.01, 0.1, 1, 10, and 100mg/mL. A total of 10 μL of tested volatile oil preloaded onto 1 cm diameter filter paper was placed in the chamber of the chosen treatment arm of the Y-tube olfactometer, and the same filter paper in another chamber of the chosen control arm was loaded with 10 μL of liquid paraffin. The choice of each adult introduced into the Y-tube stem within 5 min was recorded. The experiments were performed in a dark room at room temperature (25 ± 1 °C), and a light-emitting diode installed at 30 cm was used as the light source. In total, no fewer than 60 adults (at least 30 from each sex) were used in each treatment. After 5 insects were tested, we switched the positions of the volatile arm and the control arm, and after a total of 10 insects were tested, the Y-tube olfactometer was washed with 75% ethanol and air-dried.

### 4.11. Statistical Analysis

SPSS Statistics (version 26.0; SPSS Inc., Chicago, IL, USA) was used to analyze all the data, and the data are expressed as the mean ± standard error (SE). One-way analysis of variance (ANOVA) and least significant difference multiple comparison analysis (LSD) or the Student’s *t* test were used to determine the significant differences in the mean values. A chi-square test (χ2) was performed to detect significant differences in behavioral responses.

## 5. Conclusions

AgifCSP5 showed strong affinities for nine host-related volatiles, indicating its ability to identify and locate hosts. The results from molecular docking and site-directed mutagenesis verify that the amino acid residue Tyr75 plays a significant role in host recognition. The Y-tube olfactometer results showed that 10 mg/mL *trans*-2-nonenal, an insect-induced host volatile (HIPV), significantly attracted *A. gifuensis* females, suggesting that it plays an important role in the oviposition of parasitoids via host plant volatiles. These results will promote the biological control of wheat aphids by releasing volatiles to attract *A. gifuensis* in wheat fields and reducing the lag behind effect of artificial release of *A. gifuensis* in controlling aphid outbreaks. This study explored the recognition process of plant volatiles from the perspective of AgifCSP5, which not only lays the foundation for the development of future biocontrol attractants but could also be utilized as a molecular target for screening behaviorally active compounds for developing eco-friendly pest control strategies. However, whether there is a relatively low expression of other AgifCSPs in conjunction with AgifCSP5 to bind these screened active volatiles and whether these volatiles have good attraction to *A. gifuensis* in wheat fields still needs further study in the future.

## Figures and Tables

**Table 1:** Sequence analysis of *AgifCSPs*.

Gene	Accession Number	ORF	Length (aa)	Signal Peptide (aa)	pI	MW (kDa)
*AgifCSP1*	MK049013.1	375	124	18	9.05	12.04
*AgifCSP2*	MK049014.1	387	128	17	7.79	12.88
*AgifCSP3*	MK049015.1	351	116	20	9.24	10.84
*AgifCSP4*	MK049016.1	351	116	18	7.79	11.35
*AgifCSP5*	MK049022.1	378	125	20	4.59	12.05
*AgifCSP6*	MK049018.1	408	135	23	9.55	13.3
*AgifCSP7*	MK049019.1	396	131	19	4.66	12.85
*AgifCSP8*	MK049020.1	387	128	16	8.89	12.72
*AgifCSP9*	MK049021.1	354	117	25	8.99	10.52

**Table 2:** Binding affinities of different ligands to two mutants of AgifCSP5.

Ligand Name	Wild-Type		R24A Mutant		Y75A Mutant	
	IC50	Ki (μM)	IC50	Ki (μM)	IC50	Ki (μM)
*trans*-2-Hexen-1-al	18.08 ± 1.18	13.38 ± 0.87 a	15.03 ± 8.09	13.81 ± 7.43 a	14.59 ± 5.42	10.36 ± 3.85 a
Methyl jasmonate	20.07 ± 1.92	14.86 ± 1.42 b	8.89 ± 0.95	8.16 ± 0.87 c	29.68 ± 1.06	21.09 ± 0.75 a
Benzaldehyde	18.23 ± 0.88	13.49 ± 0.65 a	17.26 ± 3.62	15.85 ± 3.33 a	>40	-
*trans*-2-Nonenal	16.58 ± 1.78	12.27 ± 1.31 a	25.86 ± 16.38	23.75 ± 15.05 a	>40	-
Sabinene	16.37 ± 0.94	12.12 ± 0.69 a	22.37 ± 14.63	20.55 ± 13.44 a	26.23 ± 11.59	18.64 ± 8.23 a
(*Z*)-β-ocimene	17.98 ± 2.89	13.31 ± 2.14 a	18.19 ± 1.97	16.71 ± 2.73 a	30.80 ± 7.87	21.88 ± 5.59 a
*cis*-3-Octen-1-ol	17.04 ± 1.08	12.61 ± 0.80 a	18.94 ± 9.83	17.40 ± 9.03 a	22.95 ± 6.68	16.31 ± 4.75 a

Note: ‘>40’ indicates that the IC50 values could not be calculated directly from the tested ligand concentrations, and the corresponding Ki values of the ligands are indicated with ‘-’. Different letters indicate significant differences between the wild type and mutants.

## Data Availability

Data are contained within the article, Appendix A and Appendix B, or Supplementary Materials Data.

## Supplementary Materials

The supporting information can be downloaded at: https://www.mdpi.com/article/10.3390/ijms25126392/s1.

## Appendix A. Primer Sequences for RT–qPCR and Site-Directed Mutagenesis

**Gene**	**Sequence**
CSP1-F	GTTCATGCACAGCTGAAGGA
CSP1-R	AAGCCTGTCCCAATCAGTTG
CSP2-F	TCAAGTGTCTCTTGGAACAAGG
CSP2-R	CCCACTTGTCACGATCATTTT
CSP3-F	TCTTCAGGTGTATCATCT
CSP3-R	TTTAGCTCATCAGGTGTA
CSP4-F	TTGGCACAGGACCTTGTGTA
CSP4-R	GCTTTTGCAACAACAGCTTG
CSP5-F	AATATGGCTGAGGCAGTGGT
CSP5-R	TTTTTGGCTTTGGCTTCATC
CSP6-F	CCGAAGCATTGAAAACTGCA
CSP6-R	TGGCTCGTATTCCCTCAAGT
CSP7-F	TCCTGATGGACTGGAACTCA
CSP7-R	TTTACCCTCAAGTCTCATCCAA
CSP8-F	TGCAGCAAATGTAATCCAAAA
CSP8-R	CCACGTTTAGCTGCTTCTTGT
CSP9-F	GTCCTTGTGATGCTATTG
CSP9-R	CTAATATCTTTCAGGTCTTTACT
R24A-F	GATGATGCAGCAAATTCATATTACAATTGTTTTATGGGA
R24A-R	TGAATTTGCTGCATCATCATTATCAAGAATTTCTTC
Y75A-F	ATAGCTTCATGGGCTTCCGAGCACGATGAAAATGC
Y75-AR	GGAAGCCCATGAAGCTATTTTGTCAAATGCTGA

## Appendix B. Binding Data of Different Ligands to AgifCSP5

**Ligand Classification**	**Ligand Name**	**CAS No.**	**Purity (%)**	**IC50**	**Ki (μM)**	**Binding Energy (kcal/mol)**
Ketones	(*R*)-(−)-Carvone	6485-40-1	98	22.99 ± 0.91	17.02 ± 0.67	-
	Nepetalactone	21651-62-7	96	33.76 ± 6.81	25.00 ± 5.04	-
	Acetosyringone	2478-38-8	97	36.88 ± 6.77	27.30 ± 5.01	-
	6-Methyl-5-hepten-2-one	110-93-0	99	24.53 ± 1.43	18.16 ± 1.06	-
	Nepetalactone	21651-62-7	96	33.76 ± 6.81	25.00 ± 5.04	-
Aldehydes	Tetradecanal	124-25-4	97	20.61 ± 2.11	15.26 ± 1.57	-
	*trans*-2-Nonenal	18829-56-6	99.7	16.58 ± 1.78	12.27 ± 1.31	−5.2
	Benzaldehyde	100-52-7	99	18.23 ± 0.88	13.49 ± 0.65	−4.73
	*trans*-2-Hexen-1-al	6728-26-3	95	18.08 ± 1.18	13.38 ± 0.87	−4.37
	*cis*-3-Hexenal	6789-80-6	50	29.76 ± 1.55	22.03 ± 1.15	-
Alkenes	Sabinene	3387-41-5	75	16.37 ± 0.94	12.12 ± 0.69	−6.25
	(*Z*)-β-ocimene	13877-91-3	90	17.98 ± 2.89	13.31 ± 2.14	−6.07
	(1R)-(+)-α-Pinene	7785-70-8	97	22.54 ± 2.42	16.69 ± 1.79	-
	(*E*)-β-Farnesene	28973-97-9	80	25.87 ± 1.16	19.15 ± 0.86	-
	Myrcene	123-35-3	99.7	22.53 ± 2.51	16.68 ± 1.86	-
	(*S*)-(−)-Limonene	5989-54-8	99	28.36 ± 2.84	21.00 ± 2.10	-
	(*R*)-(+)-Limonene	5989-27-5	95	25.23 ± 4.06	18.68 ± 3.01	-
Esters	Methyl jasmonate	39924-52-2	95	20.07 ± 1.92	14.86 ± 1.42	−7.3
	Geranyl acetate	105-87-3	99.7	27.26 ± 4.87	20.18 ± 3.61	-
	*cis*-3-Hexenyl acetate	3681-71-8	95	24.53 ± 0.49	18.16 ± 0.36	-
	Methyl salicylate	119-36-8	99	20.63 ± 3.58	15.28 ± 2.65	-
Alkanes	Tridecane	629-50-5	99	>40	-	-
	Heneicosane	629-94-7	98	22.54 ± 2.42	16.69 ± 1.79	-
	Nonadecane	629-92-5	99.7	38.91 ± 7.44	28.81 ± 5.51	-
Alcohols	Benzyl alcohol	100-51-6	99	22.38 ± 4.58	16.56 ± 3.39	-
	Linalool	78-70-6	99.7	25.47 ± 3.46	18.86 ± 2.56	-
	1-Heptadecanol	1454-85-9	98	21.41 ± 1.07	15.85 ± 0.79	-
	*cis*-3-Hexen-1-ol	928-96-1	99	23.41 ± 5.54	17.33 ± 4.10	-
	*cis*-2-Hexen-1-ol	928-94-9	95	34.51 ± 2.09	25.55 ± 1.55	-
	*cis*-3-Octen-1-ol	20125-84-2	98	17.04 ± 1.08	12.61 ± 0.80	−5.13
	(+)-α-Terpineol	7785-53-7	97	29.38 ± 1.6	21.75 ± 1.19	-
	Leaf alcohol	928-96-1	98	27.86 ± 0.80	20.62 ± 0.59	-
Note: ‘>40’ means that the IC50 values could not be calculated directly from the tested ligand concentrations, and the corresponding Ki values of the ligands are indicated with ‘-’.						
