# Supplementary material for: Molecular Characterization of Chemosensory Protein (CSP) Genes and the Involvement of AgifCSP5 in the Perception of Host Location in the Aphid Parasitoid Aphidius gifuensis
Source: Int J Mol Sci. 2024 Jun 9;25(12):6392. doi: 10.3390/ijms25126392 (PMC11204085; doi:10.3390/ijms25126392)
Supplement: Supplementary file 1 [file ijms-25-06392-s001.zip › Supplementary data S2.pdf]

Supplementary data S2: Amino acid sequence of phylogenetic tree construction

>AconCSP1
MDGQLRALVFTMLVVALTPVSHSYQWPKPNTYMTRWDKVNLDEILESKRLLQHYFNCLMNKGPCPPDGQELKRA
LPEALKTACAKCSNSQREGAIKVIKYLREYEPKKFGILANKYDPQG
VYRHRYLESEYQSNST
>AconCSP2
MFRGTLLVLVVSLMLAAVYADDEKYSDKYDTLDIDAALADDATRNKYFNCFIGNGPCTEDAAYWKNNFPEAVVT
KCAKCTDWQKTAFDKIAAWYAENDEQAWTALMEKSIAEAKARNIPGAK
>AconCSP3
MKVISVLVLLAGLAIAAERPGENLNIDHVNYVLNNQRLLTNYIKCLLDERPCTGEVRELKKLLPELLKNGCNKCDT
SKRAIAEKVVRHLQTKRKQEWATLLAKYDPKGEYQKRYNSAQAPHA
>AconCSP4
MIFQSFILVIVGLSAIVAQELYSDKYDHINVDEILANSRLRESYLQCYLRSGPCVTADAKFFRDTFAEAVLTQCVKCT
ARQTEIFNKITDWYTKNEPEKYNMVIAKAVKFLAMNNH
>AconCSP5
MTLIRIFFILGIFFCSALSQQSSSYISSDKLDELLKDERLLNFHLKCTLGTGPCDKVGHSLKPLIPLVLRGTCRRCSPQD
VENIKKVIIFLQNKKPKELAKIYDKYGK
>AconCSP6
MGEGNCTPEGKELKKSLPDALATGCKSCSEKQKTGSEKVIRFLVNEVCHHFLLFIRYCHFHYRSGDVSVHYVFKSC
>AgosCSP1
MNILTIFCYVTVMCDTQVKPAVSAQRLQSVNQNVTPTNDGRKTIRETSSYPTRYDYIDIEAVMNNERIIKILFNCVMS
RGPCTREGLELKRIVPDAIQTECAKCNERQRKQAGKVLAHLLQYKPEYWKMLVQKFDPNNVYLRKYMADNDDD
EKLSLQKLSNDTTKKKRNI
>AgosCSP2
MAHLNLFVVLVASLIYFTSAAEEKYTTKFDNFDVDKVLNNNRILTSYIKCLLDEGNCTNEGRELKRVLPDALKTDC
SKCTDVQKDRSEKVIKFLIKNRSTDFDRLTAKYDPTGEYKKNLEKFEKERASAKPLKA
>AgosCSP4
MDSRIAVVCVVLAAFAVDQTVGAPQKDAVAASGPAYTTKYDHIDVDQVLASKRLVNSYVQCLLDKKPCTPEGAEL
RKILPDALKTQCAKCNATQKNAALKVVDRLQKDYDAEWKQLLDKWDPKREHFQKFQQFLAEEKKKGFTKF
>AgosCSP5
MHCKVLIALCCVAVYAVQASPAGTATAAVSADDEIKDFPAYMKRFDKLNVEQVLNNDRVLASHLKCFLNEGPCV
QQSRDLKRVIPVIANNGCNGCTERQMTTIKKSLNFLRTKKPTEWA
RLVKIYDPSGTKLNKFLDA
>AgosCSP6
MIKLILAIAFCVSITMTVVQTAPAKYTTKYDNVNIDEILNNDRLVASYFKCLMETGKCTPEGEEIKRWLPEAIENKCE
DCSEKQKLGSEKIIKFLFEKKNDMWKQLEAKYDPQGTYRQRYAEEAKKLNINV
>AgosCSP7
MSRSSSSVTMKVFVIAICVCAALARPEDVKVENKPAVIKSETLAAPLPTNIVKRATDTIQLDSSLPNVSEDVLDKALS
DRRFVQRQLKCATGEGPCDPIGRKIKAHAPLVLRGMCVKCSQSEIKQIQRVMSHIQKNYPKEYTKMLKQYQSGF
>AgosCSP8
MNNIIMNNSRGRYGIFSLLAVTIAAIMLVHQPATVRCADGGIITPQQQQQQTMMFTAPTGYYVSTYDHIDVGRLLRN
NKVVSGYVKCFVNEGPCTPDGKLVKAYLLPEIIRTVCGKCTPRQKDMARMVLKHIYTYRQADFEKIMQIYDTDGK
RNEILAFMNH
>AgosCSP9
MSAFCLNSFILMTMITVIVTHATFTRSTKFDDRTGIDIHLVKRDTDDVNDDENSVESDEGFFYRFTHFFQDSSDKED
DDDDEKKPDFITTFDIFKLLDEEYAMQQFYCVINEDPCDEVGMRLKATIPEEINRNCERCTSTERNNIRRILNYVKKH
YPQFWKRVEPIYKKKI
>AgosCSP10

MINTRPRKLVRCIRGVSISVAKGDDAVNAENKDDDSHLVNREEIQRYMSMMEKINIDQMLNNTRLMSNNVKCFLN
EGPCTAHLREMKKMVPMLVKDSCSSCTKEQKIMMKKAMDAVKARRPNDYEKLSKFFDPEGKYEKKFLENLNESK
>AcerCSP1
MRHNYIVILILSLLTWTYAEELYSDKYDYVNIDEILANDRLRNQYYDCFIDAGPCLTPDSVFFKSHITEAFQTQCKKC
TEIQKQNLDKLAEWFTTNEPEKWNHFVEIMIKKKDEGA
>AcerCSP2
MASAIKALLIVCALLVYTVTAETEEGQSGRSRVSDEQLNMALSDQRYLRRQLKCALGEAPCDPVGRRLKSLAPLVL
RGACPQCSPEETRQIKKVLSHIQRTYPKEWSMIVQQYAGVS
>AcerASP3
MKVSIICLVLMAAIVLVAARPDESYTSKFDDINVDEILHSDRLLNNYFKCLMDEGRCTAEGNELKRVLPDALATDCK
KCTDKQREVIKKVIKFLVENKPELWDSLANKYDPDKKYRVKFEEEAKKLGINV
>AcerCSP4
MKTILIALVAVCFLLGEVFSEDKYTTKYDNVDIDVVLNTERLLNGYVNCLLEQGPCTPDAAELKKNLPDALENECS
PCSEKQKEIADKVVQFLIDNKPEIWVVLEAKYDPTGAYRQHYLQNRVKEESY
>AcerCSP5
MKIKILLFFTILALINVKAQNDISKFLMDRPYVQKQLHCILDRGHCDVIGKKIKELLPEVLNNHCNRCTSRQVGIAN
TLIPFMQQNYPYEWQLILRRYKIMKYY
>AcerCSP6
MKIYILLFVLVTITCVIAEDYTTKYDDMDIDRILQNGRILTNYIKCMLDEGPCTNEGRELKKILPDALSTGCNKCNEK
QKHTANKVVNYLKTKRPKDWERLSAKYDSTGEYKKRYEHVLQFAKNN
>AmelCSP1
MRHNYIVILILSLLTWTYAEELYSDKYDYVNIDEILANDRLRNQYYDCFIDAGSCLTPDSVFFKSHITEAFQTQCKKC
TEIQKQNLDKLAEWFTTNEPEKWNHFVEIMIKKKDEGA
>AmelCSP2
MASAIKALLIVCALFIYTVTAETEEGQSGRSRVSDEQLNMALSDQRYLRRQLKCALGEAPCDPVGRRLKSLAPLVL
RGACPQCSPEETRQIKKVLSHIQRTYPKEWSKIVQQYAGVS
>AmelASP3c
MKVSIICLVLMAAIVLVAARPDESYTSKFDNINVDEILHSDRLLNNYFKCLMDEGRCTAEGNELKRVLPDALATDCK
KCTDKQREVIKKVIKFLVENKPELWDSLANKYDPDKKYRVKFEEEAKKLGINV
>AmelCSP4
MKTILIALVPVCFLLGEVFSEDKYTTKYDNVDIDVVLNTERLLNAYVNCLLDQGPCTPDAAELKRNLPDALENECS
PCSEKQKKIADKVVQFLIDNKPEIWVLLEAKYDPTGAYKQHYLQNRVKEESY
>AmelCSP5
MKIKILLFFTILALINVKAQDDISKFLKDRPYVQKQLHCILDRGHCDVIGKKIKELLPEVLNNHCNRCTSRQIGIANT
LIPFMQQNYPYEWQLILRRYKIMKYY
>AmelCSP6
MKIYILLFVLVTITCVIAEDYTTKYDDMDIDRILQNGRILTNYIKCMLDEGPCTNEGRELKKILPDALSTGCNKCNEK
QKHTANKVVNYLKTKRPKDWERLSAKYDSTGEYKKRYEHGLQFAKNN
>BmorCSP1
MKCLTIAALLFVAGLSIAEKYTDKYDNIDVDEILENRKLLVPYIKCVLDEGRCTPDGKELKAHIKDGMQTACAKCT
DKQKVSARKIVKHIKQHEADYWEQMKAKYDPKDEFKEIYEGFLAGQN
>BmorCSP2
MKSVILICFLGVATVVIARPKTPFDNINIEEIFENRRLLLGYINCILERGNCTRAGKDLKSSLKNVLEENCDKCSEDQR
KSIIKVINYLVSSEPESWNQLKSKYDPEGKYLIKYEAKMESN
>BmorCSP3
MNSLIAFCLFAVLAVALARPDDKYTDRYDNVNLDEVLSNSRLLQPYIKCILDKDRCAPDAKELKEHIREALETECAK
CTEAQKKGTRRVIGHLINNESKSWNELTAKYDPENKFTAKYEKELREIKA
>Bmor CSP4
MKVLIVLSCVLVAVLADDKYTDKYDKINLQEILENKRLLESYMDCVLGKGKCTPEGKELKDHLQEALETGCEKCT

EAQEKGAETSIDYLIKNELEIWKELTAHFDPDGKWRKKYEDRAKAKGIVIPE
>BmorCSP5
MNSLIAFCLFAVLAVALARPDDKYTDRYDNVNLDEVLSNSRLLKPYIKCILDKDRCAPDAKELKEHIREALETECAK
CTEAQKKGTRRVIGHLINNESKSWNELTAKYDPENKFTAKYEKELREIKA
>BmorCSP6
MKSLIVLSCLLAACLAADLSKYENFDVEPIVTSDRLLKAYINCFLDKGRCTPEASDFKKALPDTIATNCGKCTEKQK
ANVRKVIKVIQQKHSTEWEKLVKKHDPSGKHRADFDKFLLGS
>BmorCSP7
MKGFYVLCFALFAAVYCKETYSSENDDLDIEALVGNIDSLKAFIGCFLETSPCDAVSGDFKKDIPEAVAEACGKCTPA
QKHLFKRFLEVVKDKLPQEYEAFKTKYDPQGKHFDALLSAVANS
>BmorCSP8
MKTILILCALVSVVVCRPEEYYSSQYDNFDVEQLVGNLRLLKNYAKCFLDQGPCTAEGTEFKKRIPEALRTKCAKC
NPKQRHLIRTVVKAFQTKLPDLWEELAIKEDPKGQYKHEFTAFINAMD
>BmorCSP10
MKSSLFCVLVLTVVVSSSRQQSYPRNDNININAILQNDRILLGYFKCVMDRGPCTKDGKTFKRALSEALPTACARCS
NKQKAAFRTLLLAIRARSEPSFLELLDKYDPSRSNRELLYTFLATGL
>BmorCSP11
MKLTSFLLVGMAMVSAEFYSSRYDDFDVKPLVENDRILQSYTNCFLDKGPCTPDAKEFKKVIPEALETTCGKCSPK
QKQLIKTVIKAVIERHPEAWEELVNKYDKDRKFRPSFDKFINEDD
>BmorCSP12
MFMLFIISFIIVPVLKCCGTETSTYTTQYDEVDIKEIMGNERLLVAYIGCLLDKNPCTPEGKELKRNIPDALQSDCSKC
SDKQRENADAWIEFMIDNRPEDWTKLEER
>BmorCSP13
MKLLLVFLGLFLAVLAQDKYEPIDDSFDASEVLSNERLLKSYTKCLLNQGPCTAELKKIKDKIPEALETHCAKCTDK
QKQMAKQLAQGIKKTHPELWDEFITFYDPQGKYQTSFKDFLES
>BmorCSP14
MKSSLFCVLVLTVVVSSSRQQSYPRNDNININAILQNDRILLGYFKCVMDRGPCTKDGKTFKRALPEALPTACARCS
NKQKAAFRTLLLAIRARSEPSFLELLDKYDPSRSNRELLYTFLATGL
>BmorCSP16
MIEWKRFKILHFLSYLGLLVLVVVCAAQQNRPQVTDTALDEALNDKRFIQRQLKCALGEAPCDPIGKRLKTLAPLV
LRGACPQCSPQETKQIQKTLSYVQRNFPQHWAKLVRQYAG
>CcinCSP1
MTLIKIALLCLIVYTVAGQKAEEPRKPRVSDEQLNVALSDERYLRRQLKCALGEAPCDPVGRRLKSLAPLVLRGSCP
QCSPEETRQIKKVLSHIQRSFPKEWSKVVQQYAGV
>CcinCSP2
MKITLVCLIGFAAVVAVSAAPQYTTKYDNIDLKQILESDRLLNNYFNCLVNKGPCTADGQELKKALPDAIETGCKSC
NDKQKKGSDEVIRHIYKHKPEMWKVLTEMYDPERIYIKKYETEAKDLGIAV
>CcinCSP3
MRTQLLLVAVVVGVFALCQAQDISLLLNDRNYVEKQINCVVGKGSCDRIGQQIKVLLPEVLNNQCSRCSPQQAQN
ARKLVDFMKQRYPNEWRIILKRFSGRQG
>CcinCSP4
MKVAVIILVLVGCAIAAEKYTTKYDNIDLDQILKSDRLLNNYVNCLLEAGNCTPDGKELKKSLPDALANDCSKCSA
KQRDGSEKVIRFLVNKRPQIWEKLAKKYDPSGQYKIKFEGEAQKVGIKL
>CcinCSP5
MRFIPVFLACLTVAFAQEYYSSKFDNINIKEIIDNTRLFAKYKECVLQEKATRCPQEALELKRVLPEALGTLCAKCTP
SQVTKIREGLSYACKNRRVDYDEILRHVDPQGDKIVAFEQKFGKVEC
>CcinCSP6
MGERLLAFTLILCLVVIAVPKGTQGYLWPRHDTYTTRWDKVNVDDILESKRLLHYYFNCLMNKGPCPPDGHELKR
VLPEALQTACAKCTKSQIEGSVKVIRYLRQFEPGKFKQLAERYDPEGMYRKRYLEQSLDNNTA

>CcinCSP7
MIGVYVLVLAVIGCNAVYADEFYSNKYDNVNVNQILKSERLLQRYILCLLDKGSCTSDGRFFKEILPEALATNCSKC
SMKQREIVKTLTLHLMNNKPDHWREFVEKYDPDNKYRTSFLNFIMSS
>CcinCSP8
MKTFIVFFAIFGVLLAEENKKYTTKYDNIDVDVVLKTERLLNNYIGCLLNENPCTPDAAELKKNLPDALATDCTAC
SEAQKVASDKFSQYMIEERPDDWNRLENKYDPSGAYKTRYLEEKSKKSKKPN
>CcunCSP1
MTPKRSLIVVATALLVLVAGVVRAEDKKYDSKYDNLDVEAILQNDAERNIYYACFMDTGPCPNEAAIFFKGHAPEA
VVTSCRYCTQKQLEMFEKIVSWFVDNSPQEWNALIEKTINDARKQGLSF
>CcunCSP2
MKVILFVFLAFYAVAAEQLYSDQYDYVDVSKILSDDALREEYYNCYMGTSPCLTADAQYFKEILPEAALTKCVKCT
DKQKDNFQKIATWFTKNQPEKWDAYTKKAVEIYNQSQKAPE
>CcunCSP3
MSRPEINLEFVVSITLLALCIARAADEDNVPLKKPIDMAELFDKNAMKDHYPIAWTEVNTKTIIDNDRLFKKYKECL
TNEHPVSCPRMVMEFKKLIPEMIDTLCAKCLPIHIEKFKEAVEYICHRRRAEYDQVRREKDPDGAIQKKFEEQFGKV
NC
>CcunCSP5
MLRRGVYHALAIATMFLAGVVVAQDTSEESTEITTELPFQKNNRTNEFYPISWTKYNYKFIVDNERLFRKYKQCLL
VDKTTGCAHDVLQLKKIIPEVLESMCAKCLPVHVERFKEIVEYVCKKRRADYDEVRKAKDPAGLLQKKFEDKFGK
VNC
>CcunCSP6
MDKRMCWLALCWLLGGCLDKPINNEAIVNGYPWPEPGTYMTRWDKIDLNELFKSKRLMRHYFNCLVNKGPCPP
DGRELKRALPEALENGCAKCSKSQLESAIKIIRYLREFEPVKFEILANKFDPKGIYRKRYLDPTPDETNNSITDENSV
DENDQKLKRLIKR
>CcunCSP7
MKHTCAVVVLMLLLLLAIVASAQDVNILLQNKNLVSREIGCVLQRNPCDVIGKQIRGLLPEALNNGCGRCTPQQAT
NAKKLIAYMKKNYPNEWVMIAQMYGRAKAVY
>CcunCSP9
MKQLCTLVFCCVALLLAVNAAEYNSKYDNVDVDRILQNGRVLTNYIKCMLDEGNCTPDGRELKKTLPDALATGCI
KCNEKQKATADKIINHLMKRRPADWEKLLRKYDPKGEFKKRYEAQGRKI
>CcunCSP10
MATKLVFVLAICALAAVVCAKELYSDKYDNINIDAILANDSVRNEYYNCMLDFGPCVTPDAAYFKGLLGEIIITNCR
KCTDKQRYMFKQVLKHYTLKEPQKWQELVLKVLKELPKLK
>CcunCSP11
MHSYYSSRSTRRAYTLNGFYERLFLLEMFLRVCNLVYFIAGALPEALENGCAKCSKSQLESAIKIIRYLREFEPVKFE
ILANKFDPKGIYRKRYLDPTPDETNNSITDENSVDENDQKLKRLIKRHRSTIA

>CjapCSP1
MARLNCIIILISIASCVLAEELYSDQYDHIDVNNILNNDKLRDQYFNCYMETEPCLTAEAKFYRDIASEALQTKCKRC
TEKQKEIIDAVVDWYTQNKPDKWQKIVEKSLEDMKKKNAGQ
>CjapCSP2
MARLICTIAIIGIALMCVLAEEEKYEDKYDDIDVHEVLENVKLREQYYKCFMATGPCVTADQKFFSKIVSEAFQTKC
KLCTEKQKYMLDEISEWYTKNDPEKWNAFIAKTLEDMKKKAKE
>CjapCSP3
MKVLALLLIAVACALADDKYTTKFDNIDVDAILKSDRLLKNYVNCLLDKGNCTPDGKELKEHLPDALETECSKCS
EKQRTGTEKVIRFLVNKKPETWEQLKKKYDPNGEYSRRYEDEAEKRNIKA
>CjapCSP4
MKHLVVALITALSFSVVLAEDVQYTTKYDNIDVDAVINSERLLNGYVGCLLDRTPCTPDAAELKKNLPDALEHDCA
GCSEMQKNAADKISHHLIDNKPDDWRLLEDKYDPTGAYRRRYLENKSHEGGRLD

>CjapCSP5
MKFALVCLFAISTIVCVYGRPQDHYTDKFDNIDVDQILNNDRLLKRYVDCLLERSHVKCPSEALELKKVLADAMAT
DCAKCTDRQKEIARKALDFLIINKTDMWNDLKSKYDPEEKYAKKYEDRALKKEN
>CjapCSP6
MALTIKFLILVCALFTATMAAESDNSEGQQSGRSRVSDEQLNIALSDKRYLTRQLKCALGEAPCDPVGRR
LKSLVPLVLRGSCPQCSPEETRQIKKVLSHIQRSFPKEWSRIVQQYAGVS
>CjapCSP7
MDKSSLCLLALGVLAAVIAEEMYSDMFDHINPDDILPNDELRNQYYNCFMDTGPCVTEDQKYFKEHAAEAFATKC
RKCTEVQKKNVEKIVVWYTENRPQEWQAMVQKLMDDAKKLNIPFTR
>DallCSP1
MKSSVFFVLAILGAAFIAAEGGNRYADKYDSVNVDQLLGNERIYKQHLNCLLDQGQCSRQAQSLKDVLPEVLSTS
CAKCSPVQRQMARKVVGYIQKNKPDDWKLLTTKFDPQGRYTEEIRRFILSNV
>DallCSP2
MLRGAVVIALVFLSAVIAEEKYSEKYDYVDVDGILANDKQRESYYKCFAGIGPCKTAAARFFRDTLPEAIVTRCKK
CTARQSVNFDKISDWYTTNEPEKYQIIVAKAVRDIMAKSA
>DallCSP3
MRRLIFFVLIAVALAEERPMYTTKYDKFDIDSIIKNDRLFKNYIDCLMDEKPCTPEGNEFKRNLPDALETGCASCSK
AQKTMAEKFYHHVIDNRIDDWMRLENKYDPRGNYRKNYLGLDIETTTVAL
>DallCSP4
MKIAVFVLLSCLVAVISARPDKYTTKWDNIDVDQILNNDRILNNYVNCLLEEGNCTAEGRELKSVLPDALETECEKC
SRKQRDGSKKIIKFLVQNKQDLWEKLMDKYDEEKKYRGKYEDQARAEGIEIQS
>DallCSP5
MKVAFVLLAVVAVSLAKPQGYTTKYDNVDLDQILRNDRLLNNYVKCLLDEGHCTSDGKELKASLPDALATGCTK
CSEKQRAGSEKVIRYLVNERPKVWQKLAAKYDPHDEYRVKFQGEASARGIQV
>DallCSP6
MRAVVILCLLIGSVIAQKAGKYDNVDVDAILKNNRVLTQYIKCMLGEGSCTAEGRELKKVLPDALKTNCAKCDEK
QKSTAEKVINHLRSNRPNEWNRLVAKYDPQGEYEKRFEAAASAKN
>DallCSP7
MFTKVLLISLLMCAAVMGQEAEQRSRVSDEQVNIALNDPRYLKRQIKCALGEAPCDPVGRRLKSLAPLVLRGSCPQ
CSREETHQIKRVLSHIQRQFPREWSKVIKQYAGV
>DallCSP8
MEAKPSTQVLVFFLFTVVILTRDIECYTWPRRNTYMTRWDKVNLDEILQNKRLLHHYFRCLMGVGPCPPDGQELK
RVLPEALETACAKCSKSQKEGAIYVIKYLREYMPKKLEMLANRYDPDGKYRRRYYHSTSVDNNTT
>DallCSP9
MKARMALFLVGMLSLVVGIEAQDVEALLKNPEFVNFEINCMLDEGPCDLIGNSIKNVLPEALNNNCRRCTRSQARI
IRRLIDFMETAYPEQNQRIRNRYIRSPTSELADELP
>EforCSP10
MLKILSLAFLTMVFGVFVIDATYSTADPEIDFYIHNPKLIRKYLDCVEKRSTSTCGIIARRISRLIPEALFNQCRACTPD
EAAKAHKIIQFVRTYYPYDFNLIWRMYYPGAPSGQY
>EforCSP9
MSHKYLIALCFVVLVQAFAFAEEEEKYSDKYDDIDLDEVLKNDRLREQYFKCFMDEGPCNTGVIKFFKEKFPEALA
TQCKKCTEKQKAGFEKLITYYSEKEPENYQKVLEKLLNKKSA
>EforCSP8
MKSTIALVLCLVLVVAGEEQQYTSKFDHIDVDRVLKNDRLLRPYLNCLLKDMQCTPEARELKRLLPDALATKCEKC
TAKQKEGSEKVIAFLSKNKPEEWEQVLEMYDKDHIYRTKYAAEAKARGIQV
>EforCSP7
MDKRMCWLALCWWLGGCSNLDGGNDKAGLSTAGTADGYLWPKPNTYTTRWDKVNLDEILGSKRLLQHYFNCL
VNKGPCPPDGRELKRECWFRVL
>EforCSP6

MTSYKTQFALLMVVFLATTYVQAQNIDVMLRNRQLVQRQIKCVLKKAPCDAIGKQIVAQLPEALYNDCRRCKPQD
AQNSRKLLAFMQKNYPNELQQMYIIYKPQPH
>EforCSP5
MNAVLMILGLLAGMALGQDQYTTKYDNIDVEAIIKNERLLKNYVGCLLDNNPCTPEGTELKKNLPDALETNCKSC
SDIQKRISDRLTHFLIDNRPDDWALLEQKYDPTGSYKKQYLG
>EforCSP4
MKSYAIVLIMVVMITKTESQNVQMLLQNRQLVEREISCVLNRGPCDIIGNVIKSTLPEALNNNCRNCTPQQAQASQ
QIIAFMRAYYPRESQEILQLYGRRGK
>EforCSP3
MTRICLLVAFLVAGILADEEARRGLYSSEFDNLDVEAILNDDAERDKYYACLMDTGPCHSEAAVFFKDLVPEVVVTS
CKYCTPRQLEIFGKIVTWYIDNKSKEWKELVVKTIEDARKRGLLDY
>EforCSP2
MKLFAVVLLTLASFALAQQEEEKYDDKYDYLDVDGILKNDRLRQQYLDCFLETKPCVTADAIFIKKNFPEAVVTKC
RKCTEAQKMGFEKLINWMTKNDPETWRAILRKSIEDFTKKGNERRAKEKGERLRNSA
>EforCSP1
MKFAVAVVLCALASVTLAAEMPTIPSKYDSINVDMILKNDRIFRNYMKCVLENKSCSPEGRDLRMYLPDALRTRCS
NCTPKQKATAQKIIKFMMEKKKDDWKKLLEAFDKTGEIEKSFKESGGIL
>MmedCSP1
MKVAIIFLAIIAVALAATTKTYTSKFDDVDVDGILGSDRLLRNYVNCLLDRGPCTKEGVTLKEILPDALATSCESCTE
KQKTKSEKVIRHLVNNKKELWDELAVKYDPNNEYRKKYEDQA
KAKGINV>MmedCSP2
MKMFIVLMLAAVTVASVSSFVSAEAVNNKGMYSTKYDNIDINAIIKNERLLNNYVGCLMDEKPCTPDGAELKKNL
PDALASECASCSPAQKNIANVMYHHLIDNRPDLWSKLETKYDPSGGYRKRYLNQDHDQDQNEGNEEEIKSTTMAI
>MmedCSP3
MKYLGFLVIAVIFSAVSCDELYSDKYDNLNVDEALANAEVRQTYFNCFMDKGPCGEDATYWKGNFPEAIATNCKK
CTEWQKEAFDKIADWYTVHEPDNWNSFVDKMVQGARNFGDSRK
>MperCSP1
MNLLAVFCYITMMCDSQLFKRLEQPAAISQVKRIEQPAMIANRIGQPTVAPRFGQPTIAPRFGLPTIAPQVGQAAITP
QVGQAAIASRFGLPTVAPQVGQAAITPQVGQAAIASRFGLPTVAPQVGQAATTPQVGQAAIASRIGQNFQNANNSV
SPTTDGRKTTRETSSYPTRYDFIDIEAVMNNERIIKILFNCVMNQGPCTREGLELKRIVPDAIQTECAKCNERQRKQA
GKVLAHLLQYKPEYWNMLVKKFDPNNVYLKKYMADNDDDEKVSLQKLT
NDTTK
>MperCSP2
MAHLNLFVVLVASLVCFTLAEEKYTTKFDNFDVDKVLNNNRILTSYIKCLLDEGNCTNEGRELRKVLPDALKTDCS
KCTEVQKDRSEKVIKFLIKNRSTDFDRLTAKYDPSGEYKKKIEKFDSEKAAAAKH
>MperCSP4
MDSRIAVVCVVLAVFAVDQTVGAPQKDAVAASGPAYTTKYDHIDIDQVLGSKRLVNSYVQCLLDKKPCTPEGAELR
KILPDALKTQCVKCNATQKNAALKVVDRLQRDYDKEWKQLLDKWDPKREYFQKFQQFLAEEKKK
GVVKF
>MperCSP5
MNCKVLIALCCVAVYAAHASPAGAATAAAASADEEIKDFPAYMKRFDKLNVEQVLNNDRVLASHLKCFLNEGPCV
QQSRDLKRVIPVIANNGCNGCTERQMTTIKKSLNFLRTKKPVEWARLVKIYDPSGTKLNKFLDA
>MperCSP6
MNTLLLAVALCIAITMTVVQTAPAKYTTKYDNVNIDDILNNDRLVASYFKCLMETGKCTPEGEEIKRWLPEAIENKC
ENCSEKQKIGSEKIIKFLIEKKNDMWKQLEQKYDPQGLYKQRYSEEAKKLNLDV
>MperCSP7
MDRSSSSVTMKVFVIAVCVCAALARPEDSKVENKPAAVKSETLAAPLPTTIVKRATPQVVSTQQGASLPNVSEDVL
DKALSDRRFVLRQLKCATGEGPCDPIGRKIKAHAPLVLRGMCVKCSQSEIKQIQRVMSHIQKNY
PKEYTMMLKQYQSGF

>MperCSP8
MTNNNMNSPRCRPEIFSLLAVAAIATVLVHQPSTVHCADAGVYPPQQQQQEATMFTAPSGYYVSTYDHMDVGRLL
RNNKVVAGFVKCFTNEGPCTPEGRLAKAYLLPEIIRTVCGKCTPRQKDMARLVIRHIYTYRRGDFDKIMQIYDTDG
KKNEIIDFMNQK
>MperCSP9
MTSFCLNSVILMTITTVIVAHAASTGMTAFNNRSGSDIHMAQRDYNENKADKAEGFFFTITNFFSRRKHDDDKPDFI
TTFDIIRLLDEKYAMKQFYCVINKEPCDATGLRLKATIPEEINNDCERCTATETSNIRRILNYVKKHYPEFWDRVEPIY
RNNMTA
>MperCSP10
MVSKLFVSVFVLMSVVGVSYSVTEGDDDAAKVADKDLHPVNQEELKKFLSMMEKVDIDQILNNNRLMSNNVKC
FLNEGPCTGQLREMKKMVPMLVKDSCSSCNKEQKNMMKKAMDAMKARRPNEYEQISKFFDPEGKYEKKFLENL
NESK
>MpulCSP1
MYCPILLIILASLVITIHADELYSDKYDGLDIDGILANEELRKQHENCYMDRGPCDDAAEFFKSHFPEVVATACSKCT
EWQSQAFDKIADWYNKNDEATWNAFVANNMELAKTMNIR
> MpulCSP2
MSERVRRIRNLQTIAIGINVAVAQSRSISFSTSSQYNTRVNMKVAVVFIIAVVAVVMGAKYTTKYDNVDLDQILKSNR
LLNNYVDCLLSKRTCTPDGKELKENLPDALKTECAGCSEKQKAGSEKVIRYIVNQRPDLWEQLAKVYDPNNEYRV
KFQDKAEEKGIKI
> MpulCSP3
MSERVRRIRNLQTIAIGINVAVAQSRSISFSTSSQYNTRVNMKVAVVFIIAVVAVVMGAKYTTKYDNVDLDQILKSNR
LLNNYVDCLLSKRTCTPDGKELKENLPDALKTECAGCSEKQKAGSEKVIRYIVNQRPDLWEQLAKVYDPNNEYRV
KFQDKAEEKGIKI
> MpulCSP4
MSRKIVELLCSLIIIHINVISAVDHVHFSPEILLRNERLLARFVDCLVDEGPCLGPIAKFKKEIPKMMETQCARCQEQI
KVLSAKVMQHIREHKPEKWKKIQEIYDPDHKHQEKLDGLLDDYQ
> MpulCSP5
MALLIKIFLLSVIACATHGQESTGRGISQFHISDEQINMALNDRRYLRRQIQCVLGEAPCDPVGRRLKSLAPLVLRGS
CPQCSKDESRQIKKVLAHVQRTFPKEEWTKIIKQYAGVLSKN
> MpulCSP6
MRNQTVIFVALVSILSMIINTSNARDFDDLQRDGTYMIQVIDCVLGTAACDEYGEKVKALIPEALNNNCKRCNPEQ
KRKFKLMSAFMKMAYPDQWQQIQEKYYSNRYIHADY
> MpulCSP7
MTNHFIIYIHSSCVRIFIINLYDYFICFLLFFILDRFPEAVVTNCRKCTRRQSENFDKLTDWYTKHEPEKYNAIVGMAL
KKLMKKS
> MpulCSP8
MYCPILLIILASLVITIHADELYSDKYDGLDIDGILANEELRKQHENCYMDRGPCDDAAEFFKSHFPEVVATACSKCT
EWQSQAFDKIADWYNKNDEATWNAFVAKNMELAKTMNIR
>SaveCSP1
MAQLNLFVVLVASLVCFTLAEEKYSTKYENFDVDKVLNDDSLLTSYINCLLDEGNCTEEGQALKRVLPDALKTNC
GKCTDTQKMKIEKILKFLMKNRSTDFDRLTAKYDPSGEYKKKLEKFSA
>SaveCSP2
MDSRIAVVCVVLAVFAVDQTVGAPQKDALAAGSPTTYTNKYDHIDIDQVLASKRLVNSYVQCLLDKKPCTPEGAE
LRKILPDALKTQCAKCSATQKNAALKVVDRLQKDYDKEWKQLLDKWDPKREQFQKFQQFLTEEKKKGVVKF
>SaveCSP3
MNCKVLIALCCVAVYAAQANPAGAATATAADDEIKDFPAYMKRFDKLNVEQVLNNDRVLASHLKCFLNEGPCVQQ
SRDLKRVIPVIANNGCNGCTERQMTTIKKSLNFLRTKKPVEWARLVKIYDPSGTKLNKFLDA
>SaveCSP4
MARSSSTSVTMKVFVMAVCVCAALARPEEAKMENKPTAVKSETLAAPLPTTIVKRATPQVVSIQKDASLPNVSEDV

LDKALSDRRFVQRQLKCATGEGPCDPIGRKIKAHAPLVLRGMCVKCSQSEIKQIQRVMSHIQKNYPKEYTKMLKQ
YQSGF
>SaveCSP5
MNLLAIFCYITMMCDSQFRRLEQPTAIPQVKRIEQPATIATRIGQATIAPRFGQPTVAPRFGQPTIAPRFGQATAAPQTG
EAAIGPRIGQTFQNVNDSVSPTTDGRKTTRETSSYPTRYDFIDIEAVMNNDRIIKILFNCVMNQGPCTREGLELKRIVP
DAIQTECAKCNERQRKQAGKVLAHLLQYKPEYWNMLVKKFDPNNIYLRKYMADNDDDEKLSLQKLSNNTTK
>SspCSP1
MAFINFTILTIFSIILIAFAEKIELYSNKYDHIDADTILSNERLRNQYLNCYLGSGSCITPDARFLRDTFPEALVTKCKRC
TQKQHILFEKITLYFTEKEPDTWNKILLKAIENSRKRH
>SspCSP2
MNRVSFVALLALATIAFVCGQEEELYSDRYDYIDADDILKNERLRNQYYKCFYGSGPCVTADAKFFKNNFGEAIVT
KCKKCTQKQISNFDKMIVWYNENAPDQWEALVKKLVENAQENAN
>SspCSP3
MKEIIIFIAIIAVALAAEKYPSKYDDVDVDRILQNSRVLTNYIKCMLDEGSCTAEGRELKKTLPDALQSGCSKCNEKQ
KQTAEKVINHLRTKRPRDWDRLVAKYDPQGEYKKRYENLTTKKV
>SspCSP4
MVNIISSILLFSFVFLQYSFAEEMYSDKYDNIAIEDILNNDKVREEYYNCFMDTGPCVTEDAAYFKGNFVEAMATQC
KKCTQKQQENFEKVIVWYTENQPEKWQTLIQKALEDAKKLNIPMEATS
>SspCSP5
MASTIKVVCVICVFVTLALADGSEERGGKSRVSDDQLNMALSDDRYLRRQLKCALGEAPCDPVGRRLKSLAPLVL
RGSCPQCSPEETRQIKKVLSHIQRSFPKEWSKIVQQYAGV
>SspCSP6
MKVAIVVLAVISCALAQRYTTKYDNIDLDQILRSERLLNNYVNCLLDAGNCTPDGKELKKSLPDALASGCSKCSEK
QKEGSEKVIRFLVNERPQVWDKLAKKYDPTGEYKLKFQGQAQTHGIQI
>SspCSP7
MKSIIVIFTVFAIVFAQESTPEYYTGKWNDLNTHDIVDNARLFKKYKQCIMAETNTGCPQEVIELKKVLPEALETVC
AKCSPVQVEKIRDTLKYVCEKRKTDFDDILKHIDPEGTHRPKFEEKFGNLGC
>SspCSP8
MQFKSVILIVGLVIISISAEEQYTSRFDTINVDEILKSERLLNNYFKCLMDRGRCTPEASELKKVLPDALETDCMKCT
NLHKKMARKVIDYIVKNKNEMWQELVGKYDPKGTYRTKFEKDAVAAGINIQA
>SspCSP9
MAQHLLLVLLVVLAVARCVFTAPEDTTTDNKYTTKYDQVDVDAIVRNERLVNSYVGCLLDRNSCTPDAAELKKNL
PDALQTACVSCSEAQKDVADKFSQFLIDHKPDQWKLLEEKYDPDGEYKKRYLNDA
>SspCSP10
MEFGVILIFIISCGLSFGSDYYSDVYDKIDVDSILASDRLFNQYISCLLDKGPCTADGRSLRRIIPEAISTRCEKCNEKQ
KSTTKKVLVHLKEKKPDVW
>TdenCSP1
MKAVCVMLFCCMSVALAANAYTNKYDNVNVDQILNSPRLLQSYMKCMLDEGNCTPDGRELKRTLPDALATGCT
KCNEKQKAVAGKVINFLMTKKANDWARLLAKYDPNGEFERRYRAQGNRIFAN
>TdenCSP2
MSKGPCPPDGRELKRVLPEALATGCAKCTKKQIEAAVKVIKYFREFEPERFELLANVYDPHGIYRRKYFDNSLDND
VITSNSLTGERNRRMAAVAAAAAGLHSDNHPRKRLIGQ
>TdenCSP3
MANRFVLALVLCTLVAGCLADEEKYSDKYDYIDVDNILGNERIRNQYFNCFLDYAPCLTADAKFFRDHFPEALVTK
CKKCTEKQKEAFEKLVLYYTEKEPEKWRAALTKAIAESGKKRAKN
>TdenCSP4
MAGLRYLIACLAVLSTIATTSALDPNAFLRMDAVLADPALFQTYLSCFVDEGECPPDGQMLKKLLPELIATRCNKCT
ENERRTACNAIMMLEQPQYAREWQKLQQIYDPRGVNYAYLNRFKSACASVDYA
>TdenCSP5

MKATLAMVLCMAVVAVVMGADEVKYTTRFDSIDIEQVLHNDRILKSYINCLLKDAACSPDARELKRLLPDALATN
CEKCSEKQKAGAEKVISFLAKNKPETWTEILAKYDKDNVYRTKYAEQAKKLGVPV
>TdenCSP6
MKALLSYVAVFGLLLLLLLTTSGAEESKYTTKYDDLDIDSIIKNERLLNSYVGCLLDRKPCTPDATELKANLPDALA
NECSRCSERQRQISDKLSHHLIDHRPEDWQQLEAKYDPTGTYRRVYLSDRQRLKAAVATTDNTDVGPAAASGA
>TdenCSP7
MERKSVLSIILVVFICAVLTRADEDSREWILYPTTHDDFDINALLSSDQDRNSWYDCFMHKSDCPSDTAKFFKERLSE
AVMTSCKFCTEKQLQIFQKFVLWYAQNDPTAYGVLLHQMMAEAEHKGIDTIM
>TcCSP7L
MKTLVLVLFVAVLSVVFAADKYTTKYDNIDLNQILKSDRLLKNYVNCLLDRGKCSPDGQELKNNLADALQTSCSK
CSQRQKDGSRTIIRYLIKNKRDWWNELEAKYDPTGIYKNKYADELKAEGIVL
>TcCSP7R
MLFTVFLVLTCAHVVFLEEYVIPDNIDIDDILSNERLLKNYVNCLLDKGRCTPEGKKLKSTIPEALSTDCAKCNEKV
KANVRKVLHHLIDNKPDMWKQLEAKYDPSGEYRSKYKDELEKNGIHV
>TcCSP7A
MKFFIAFLMLLGAVWCEQYTTKYDNINVDEILASERLLKNYFNCIMDRGACTPDADELKRVLPDALKSDCAKCSE
KQKEMTKKVIHFLSHNKQQMWKELTAKYDPDGIYFEKYKDKFDS
>TcCSP7G
MYSYLIPLYLFLFVHYGWSEDTTHKYTTKYDNIDLENVVKNERLLKSYVDCLLEKGRCSPDGLELKKNMPDAIET
DCSKCSEKQKEGSDFIMRYLIDNKPDYWKALEAKYDPDGTYKKRYFESQKDEVSKVEA
>TcCSP7Q
MFKVLFVVFACVQAYVYAEEYTVPQNIDIDEILKNDRLTKNYLDCILEKGKCTPEGEELKKDIPDALQNECAKCNE
KHKEGVRKVIRHLIKNKPSWWQELQEKYDPKGEYKSRYNHFLEEEGLN
>TcCSP7P
MTAIVFLLALACLKTYVSSQEYLVPQNIDVDEILKNDRLTRNYLDCVLGKGKCTPEGEELKKDIPEALQNGCAKCN
EKHKEGVRKVIHHLIENKPNWWQELESKFDPQGEYKKKYDELLKKEGLAN
>TcCSP7E
MIPLIAIAGILAVSAAPAEFYESRYDHLDVESILNNRRMVNYYAACLLSKGPCPPQGVDLKRVLPEALQTNCAKCTE
KQRTAAYRSIKRLKKEYPKIWEQLRAVWDPDDVFIRKFETSFESGKPSGVISTNTSPPSPILSNRFGENEEADAASNVI
SSTPLPPTTSTTTRTTLTTKFTTKPSTKPTNKPVVVTKPPQAPPFATVGANLQATVSFGTNLVGGIVRSLGTLGSRVVE
SGTKLANMVISAAIRP
>TcCSP3A
MKIIILAVLIATAVAATYDVYPTKYDNVDIDAILHNKRLFDNYLQCLLKKGKCNEEAAILRDVIPDALITGCRKCNDH
QKVSVEKVIRFLIKERNSDWQQLISVYDPKGEYQTQYAHYLEKI
>TcCSP7O
MIFKIHFLVFGALLTYVSSVEYLILREIDTILKNDQMTRNYLDCVLDKGKCTKEAEKLKKGITETMKNGCVKCEQK
QKEDVHKVFQHLMIHRPNWWHELETKFNPHHEIKLQHLHQSKFNPHEEVKLQHLHQFPHHDFLEREGFIR
>TcCSP7I
MKLFVINFILMSLVYMSFGASVPYETVDIDKLLADDKMVTEYMACLRGEGPCNPAEKDLEEHIPLVLGNYCADCN
DKQKNFVIKLATFVIKNRFDEWRQVQKRFDPDLSHADDFNKFILGS
>TcasCSP1
MFMDCISRIRYIVRRNISKSIKIKLCSKSWPEFNRLKGFKEERIMKFLLCVTLLCCVGFTLGQLCPQDAAVDTKLRDG
RFIKKQVDCILSKGKCNGLGKKLKRKVPEVLRGLCPKPCKPCDQKQIKKIVQVMQEKYPREWNEIITTYNG
>AgifCSP1
MRSIFVIIFIAICVVVAAEEKLSSKYDDIDVDKILQNNRVLTNYIKCILGEGSCTAEGRELKKTLPDAIRSQCSKCDAK
QKSMVNKVINHLITKRPTDWDRLAKKFDPMGEYKKSFEAQAGKTA
>AgifCSP2
MKIAILFLAVMVAVVTAKPANQYTNKYDDINLDEILGNKRILNNYIKCLLEQGSCTPEGTELKMRLPDALETGCSKC
NEKQKAGAKKMIEHLIKNDRDKWDKLMEKYDPESKYRTKFEDELKTLSVSA

>AgifCSP3
MGHLFKVLLISLAFAVIVMGADDESESRGFSSGVSSGQIEAALKNDRYLKRQLQCALGTQPCDAVGKKLKELAPQV
LRNNCVKCTPDELNQIRKVLITIQNRFPKEFGMILRQYRI
>AgifCSP4
MMREIIIFALFGLSAVFANELYSNKYDYVEVDDILANPRQREQYYKCFVGTGPCVTADAKFFQEKFPEAIVTKCSKC
TPKQLTSFDKLTDWYTTNEPEKYQAVVAKALERISRKSG
>AgifCSP5
MKCLIIFFVTILIIVKLINCQEKYSDKYDSVMNVEEILDNDDARNSYYNCFMGTSSCPNEAAEYFKGNMAEAVVTSC
SRCTEWQISAFDKIASWYSEHDENAWNAFVTKFIDEAKAKNIVTPSRK
>AgifCSP6
MYERTCVLIFILLFTILPSYINCNMWPKKDHYNTRWDKMNLDKILENKRLLRYYFNCLMSKGPCPPDGQVLKKNLP
EALKTACEKCSKSQKDGGIKVIKYLREYEPKIFEILASKYDPKGTYRRRYLKSEADNTT
>AgifCSP7
MKIIISLLYFIFFAAGICADNSQMYSTKYDNIDIDEIIKNDRLVNNYVGCLMEEKSCTPDGLELKKNLPDALKNDCAS
CSDAQKMIAEKMYHYLIDNKINDWMRLEGKYDPDGIYRNHYLGIDGLSTTASI
>AgifCSP8
MKVAILLLGFFAVALGHPQSTYTTKYDGVDIEMILRNPRLLNNYVNCLLDKGKCTPDGTELKTLLPDAILSECSKCN
PKQKEGSERVITFLVKEKPDIWAKLAAKYDPQNTYRVKLQQEAAKRGITLP
>AgifCSP9
MEFSYKILLLSFVVTVLTSCSSLHADEVTEKPSVTDEQLSQALNDQRFLKFQIACALNKGPCDAIGIRLKKQVPRVM
LGLCPECSSKDLKDIRKFIMHFQTKFPKEFKELTQKFVAG
